# Toxicogenomic analysis of *Caenorhabditis elegans *reveals novel genes and pathways involved in the resistance to cadmium toxicity

**DOI:** 10.1186/gb-2007-8-6-r122

**Published:** 2007-06-25

**Authors:** Yuxia Cui, Sandra J McBride, Windy A Boyd, Scott Alper, Jonathan H Freedman

**Affiliations:** 1 Nicholas School of the Environment and Earth Sciences, Duke University, Durham, NC 27708, USA; 2 Laboratory of Molecular Toxicology, National Institute of Environmental Health Sciences, NIH, Research Triangle Park, NC 27709, USA; 3 Laboratory of Environmental Lung Disease, National Heart, Lung, and Blood Institute, Bethesda, MD 20892, USA; 4 Department of Medicine, Duke University Medical Center, Durham, NC 27707, USA

## Abstract

**Background:**

Exposure to cadmium is associated with a variety of human diseases. At low concentrations, cadmium activates the transcription of stress-responsive genes, which can prevent or repair the adverse effects caused by this metal.

**Results:**

Using *Caenorhabditis elegans*, 290 genes were identified that are differentially expressed (>1.5-fold) following a 4 or 24 hour exposure to cadmium. Several of these genes are known to be involved in metal detoxification, including *mtl-1*, *mtl-2*, *cdr-1 *and *ttm-1*, confirming the efficacy of the study. The majority, however, were not previously associated with metal-responsiveness and are novel. Gene Ontology analysis mapped these genes to cellular/ion trafficking, metabolic enzymes and proteolysis categories. RNA interference-mediated inhibition of 50 cadmium-responsive genes resulted in an increased sensitivity to cadmium toxicity, demonstrating that these genes are involved in the resistance to cadmium toxicity. Several functional protein interacting networks were identified by interactome analysis. Within one network, the signaling protein KEL-8 was identified. *Kel-8 *protects *C. elegans *from cadmium toxicity in a *mek*-1 (MAPKK)-dependent manner.

**Conclusion:**

Because many *C. elegans *genes and signal transduction pathways are evolutionarily conserved, these results may contribute to the understanding of the functional roles of various genes in cadmium toxicity in higher organisms.

## Abstract 2

Global analysis of the transcriptional response to cadmium exposure in *Caenorhabditis elegans *reveals roles for genes involved in cellular trafficking, metabolic processes and proteolysis, and for the signaling protein KEL-8.

## Background

Cadmium is a persistent environmental toxicant that is associated with a variety of human diseases. Target organs of cadmium toxicity include kidney, testis, liver, prostate, lung and tissues, including muscle, skin and bone. Cadmium has also been classified as a category 1 human carcinogen by the International Agency for Research on Cancer [1]. In addition, cadmium exposure is associated with teratogenic responses, including fetal limb malformations, hydrocephalus, and cleft palate [2-5].

At low levels of exposure, the toxicological effects of cadmium are prevented by the activation of intracellular defense and repair systems, namely the stress response. Cadmium-induced expression of stress-responsive genes has been reported in a variety of species [6-10]. Cadmium can activate transcription of many stress-responsive genes, including those that encode metallothioneins, glutathione-S-transferases (GSTs) and heat shock proteins, all of which play important roles in the resistance to metal toxicity or cellular repair. The emergence of microarray technology has enabled genome-wide investigations of gene regulation, and the subsequent identification of genes that were not previously associated with responses to cadmium exposure. For example, treatment of HeLa cells with cadmium affected the expression of more than 50 genes, out of 7,075 genes that were examined [11]. Exposure of the human T-cell line CCRF-CEM to cadmium altered the mRNA levels of more than 100 genes in a dose- and time-dependent manner [11,12]. The results obtained from these and other studies provide valuable knowledge on the ability of cadmium to alter gene expression [13,14].

In most cases, the relationship between cadmium-induced changes in mRNA levels and the biological consequence of the alteration has not been established. Only a few cadmium-responsive genes have been tested for a role in the resistance to cadmium toxicity. Mammalian metallothioneins and the *Caenorhabditis elegans cdr-1 *genes are highly cadmium-inducible. Inactivation of both MT-1 and MT-2, in MT-1/2 double knockout mice, or inhibition of *cdr-1 *by RNA interference (RNAi) in *C. elegans *results in hypersensitivity to cadmium [15-17]. These results confirmed the important roles of these proteins in the defense against cadmium toxicity.

In the present study, we utilized whole genome *C. elegans *DNA microarrays to monitor global changes in the nematode transcription profile following cadmium exposure. Bioinformatic analysis of Gene Ontology (GO) and protein interaction networks were used to identify potentially novel pathways involved in the cadmium defense response. The biological role of the cadmium-responsive genes and the cognate pathways in the defense against cadmium toxicity were studied by inhibition of gene expression using RNAi. Genes and pathways previously associated with cadmium exposure were identified, confirming the efficacy of the study. In addition, genes and pathways not previously associated with cadmium exposure were discovered.

## Results

### Effects of cadmium on the transcription of stress-responsive genes

**Figure 1:**
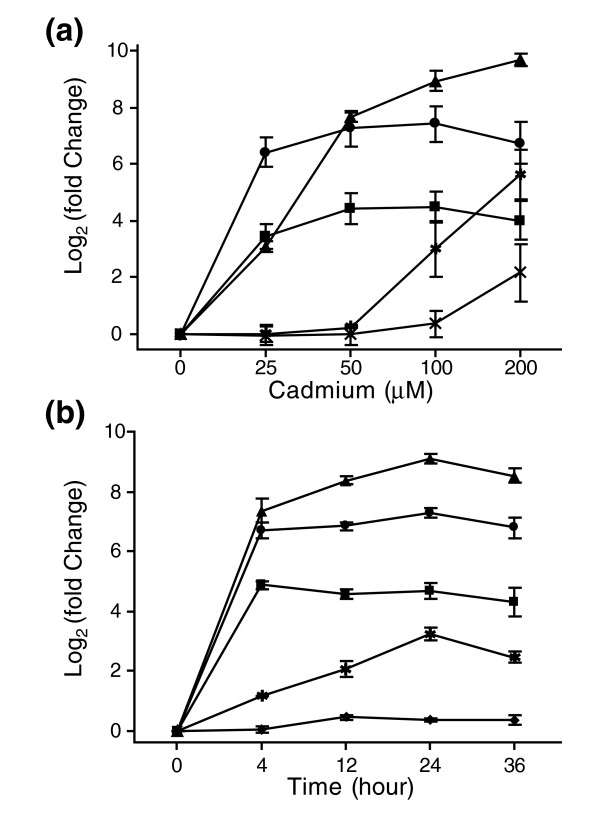
Effects of cadmium on the transcription of stress-responsive genes. Total RNA was extracted from non-treated or cadmium-treated *C. elegans*, and mRNA levels of cadmium-responsive (*cdr-1 *(triangle), *mtl-1 *(square), *mtl-2 *(circle)) and general stress-responsive (*gst-38 *(asterisk), *hsp-70 *(cross); T24H10.4 (diamond)) genes were measured with qRT-PCR. All measurements were normalized to the mRNA level of *mlc-2*. Fold change was normalized to the mRNA levels observed in non-exposed nematodes. Results were displayed in mean log_2_fold ± SE (*n *= 3). **(a) **The effect of cadmium concentration on mRNA levels following 24 h exposure. **(b) **The effect of exposure time on mRNA levels following exposure to 100 μM cadmium.

To determine the optimal conditions that affect cadmium-responsive transcription, quantitative real-time PCR (qRT-PCR) was performed to assess the effects of different cadmium concentrations and exposure times on the expression of selected stress-responsive genes. The level of expression of three *C. elegans *cadmium-responsive genes, *cdr-1*, *mtl-1 *and *mtl-2*, significantly increased at all cadmium concentrations following a 24 h exposure (Figure 1a). However, the levels of expression of the two general stress-responsive genes, *gst-38 *and *hsp-70*, were induced only at concentrations greater than 50 μM (Figure 1a).

The time course of gene induction in response to 100 μM cadmium was also examined. The expression of cadmium-responsive genes was maximally induced after only 4 h; in contrast, the general stress-responsive gene *gst-38 *reached its highest level of expression after 24 h (Figure 1b). The *C. elegans *homolog of human *jun*, T24H10.7, did not respond significantly to cadmium exposure. Based on these results, subsequent microarray experiments were performed using *C. elegans *exposed to 100 μM cadmium for 4 and 24 h.

### Microarray analysis of cadmium-responsive transcription

**Figure 2:**
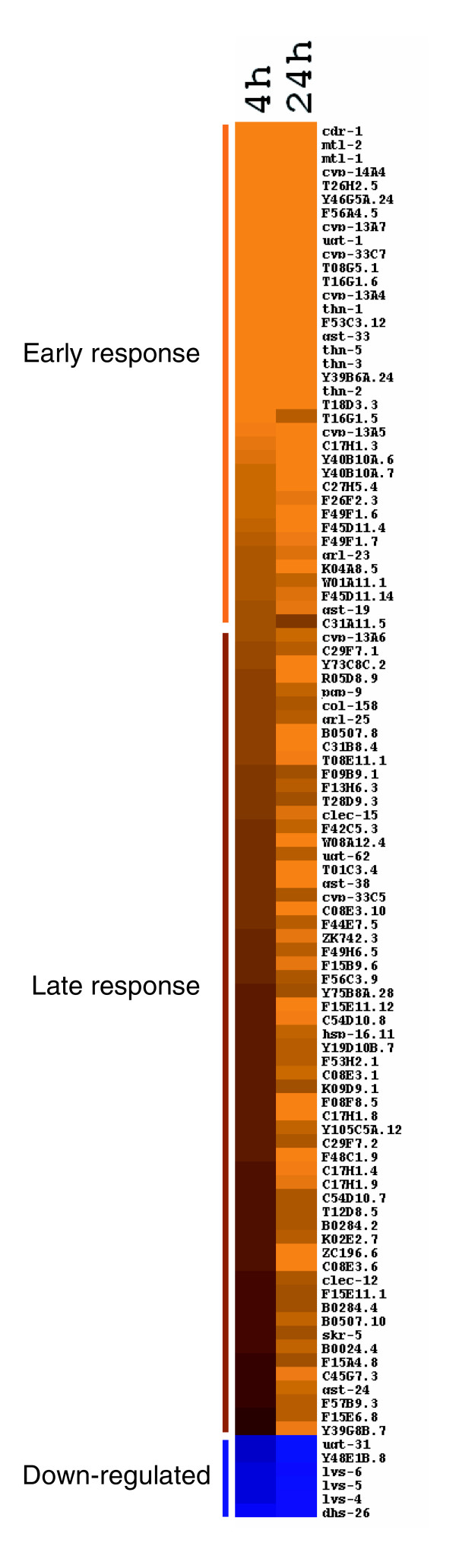
Heat map of cadmium-responsive genes. Cadmium responsive genes (≥2-fold) based on decreased expression (blue) or increased expression (orange) relative to non-treated *C. elegans*. Brighter shades of color correspond to greater fold changes in expression.

There were 37 and 95 genes significantly up-regulated after 4 h and 24 h exposures to cadmium, respectively; 6 genes were significantly down-regulated following a 24 h exposure (fold-change ≥2, *p *< 10^-6^) (Table 1, Figure 2). The genes whose levels of expression significantly changed were clustered into three groups: group 1, early response genes; group 2, late response genes; and group 3, down-regulated genes. The early response group includes *cdr-1*, *mtl-1*, *mtl-2*, and phase I and phase II metabolism genes. The levels of expression from the microarray study of *cdr-1*, *mtl-1*, *mtl-2 *and *gst-38 *were consistent with the qRT-PCR results.

**Table 1:** Genes whose expression changes following 4 h or 24 h cadmium exposure

Gene name	CGC gene name	4 h exposure		24 h exposure	
			
		Fold change	*P *value	Fold change	*P *value
**Up-regulated genes (early response group)**					
F35E8.11	*cdr-1*	73.4	1.00E-43	111.4	3.10E-43
T08G5.10	*mtl-2*	28.7	1.70E-38	31.7	<1.0E-43
K11G9.6	*mtl-1*	17.1	6.28E-40	15.0	4.71E-39
R04D3.1	*cyp-14A4*	14.9	5.32E-37	32.4	4.02E-34
T26H2.5		10.1	6.57E-27	15.2	2.88E-38
Y46G5A.24		7.4	1.32E-37	18.3	6.16E-37
F56A4.5		6.5	1.96E-29	11.8	1.32E-32
T10B9.10	*cyp-13A7*	6.3	2.57E-26	6.9	4.00E-26
AC3.7	*ugt-1*	5.4	4.16E-42	8.2	3.46E-37
F41B5.2	*cyp-33C7*	5.3	6.86E-30	6.9	5.18E-42
T08G5.1		4.9	1.20E-27	6.8	4.04E-32
T16G1.6		4.5	1.16E-41	6.1	3.91E-36
T10B9.1	*cyp-13A4*	4.3	6.38E-40	7.1	1.12E-35
F28D1.3	*thn-1*	4.0	7.88E-34	9.9	1.50E-42
F53C3.12		3.9	1.07E-31	5.7	2.12E-40
C02A12.1	*gst-33*	3.8	2.57E-33	7.8	2.15E-33
Y59E9AR.4	*thn-5*	3.7	2.50E-35	8.5	4.87E-42
F28D1.4	*thn-3*	3.6	2.69E-23	14.3	6.80E-33
Y39B6A.24		3.4	7.06E-31	11.4	4.48E-42
F28D1.5	*thn-2*	3.4	3.45E-30	3.7	3.51E-35
T18D3.3		3.3	2.18E-31	4.3	1.45E-35
T16G1.5		3.0	1.65E-30	2.2	1.02E-29
T10B9.2	*cyp-13A5*	2.9	3.11E-36	4.4	1.22E-34
C17H1.3		2.7	5.00E-17	4.0	7.58E-25
Y40B10A.6		2.6	2.99E-20	3.8	6.72E-23
Y40B10A.7		2.4	1.41E-20	3.5	1.59E-22
C27H5.4		2.4	1.90E-27	4.1	1.52E-32
F26F2.3		2.4	1.77E-14	2.7	1.66E-19
F49F1.6		2.4	6.64E-33	3.4	2.19E-38
F45D11.4		2.3	1.52E-22	3.3	6.57E-31
F49F1.7		2.2	2.93E-20	2.8	6.66E-24
E02A10.2	*grl-23*	2.1	2.47E-07	2.6	3.93E-19
K04A8.5		2.1	1.04E-25	3.0	2.50E-23
W01A11.1		2.1	2.01E-28	2.3	1.28E-31
F45D11.14		2.1	9.71E-09	2.6	2.43E-14
F37B1.8	*gst-19*	2.0	8.18E-24	2.7	2.40E-27
C31A11.5		2.0	7.33E-24	1.7	3.35E-18
					
**Up-regulated genes (late response group)**					
F08F8.5				7.1	1.06E-28
B0507.8		1.8	4.14E-09	5.6	2.51E-32
C08E3.6				5.2	2.02E-27
F35E8.8	*gst-38*	1.6	3.05E-20	5.0	1.41E-28
C31B8.4		1.8	5.34E-09	4.3	2.57E-37
C17H1.8				4.0	5.83E-19
T01C3.4		1.6	3.18E-07	3.7	3.88E-32
F15E11.12				3.4	1.88E-10
W08A12.4		1.6	2.66E-12	3.3	2.54E-27
F48C1.9				3.3	2.97E-14
R05D8.9		1.8	9.43E-18	3.2	4.16E-25
ZC196.6				3.0	1.18E-26
C08E3.10		1.6	2.44E-08	3.0	1.58E-30
Y73C8C.2		1.8	1.45E-22	3.0	3.27E-28
T08E11.1				2.9	5.78E-24
C54D10.8				2.9	3.96E-24
C17H1.4				2.9	5.74E-21
Y39G8B.7				2.8	1.55E-26
C45G7.3				2.8	7.31E-23
F15B9.6		1.5	2.29E-17	2.7	4.03E-33
ZK742.3		1.5	9.27E-14	2.7	1.04E-24
C17H1.9				2.7	8.10E-18
T07D10.4	*clec-15*	1.7	6.03E-10	2.6	1.51E-22
C08E3.1				2.4	1.12E-19
F37B1.1	*gst-24*			2.4	1.02E-18
T10B9.3	*cyp-13A6*	1.9	2.08E-25	2.4	5.68E-25
C47A10.1	*pgp-9*	1.8	1.09E-20	2.3	3.32E-21
F42C5.3		1.6	1.37E-11	2.3	3.76E-20
B0024.4				2.3	9.01E-17
B0507.10				2.3	2.11E-23
Y105C5A.12				2.3	1.06E-19
T27E4.2	*hsp-16*.*11*			2.3	9.06E-30
ZK643.8	*grl-25*	1.8	7.23E-07	2.2	2.62E-19
F15E6.8				2.2	8.03E-23
F13H6.3		1.7	3.31E-25	2.2	6.26E-31
K02E2.7				2.2	1.25E-22
F57B9.3				2.2	1.18E-19
Y19D10B.7				2.2	2.44E-13
F49H6.5		1.5	1.18E-09	2.2	1.19E-16
C29F7.1		1.9	2.15E-24	2.2	3.55E-30
F44E7.5		1.5	6.49E-21	2.2	3.59E-29
M88.1	*ugt-62*	1.6	1.06E-23	2.2	2.78E-34
F53H2.1				2.2	2.47E-23
B0284.2				2.1	5.41E-26
C54D10.7				2.1	2.83E-21
F56C3.9		1.5	3.21E-13	2.1	4.20E-23
T27F6.2	*clec-12*			2.1	1.45E-18
D2023.7	*col-158*	1.8	6.26E-07	2.1	6.44E-15
C29F7.2				2.1	1.41E-30
T12D8.5				2.1	5.12E-22
F41B5.3	*cyp-33C5*	1.6	8.21E-20	2.1	1.21E-24
F15A4.8				2.0	2.08E-22
T28D9.3		1.7	3.40E-24	2.0	7.68E-30
F09B9.1		1.7	8.66E-20	2.0	1.63E-32
Y75B8A.28				2.0	3.06E-20
F15E11.1				2.0	3.41E-11
B0284.4				2.0	4.57E-13
F47H4.10	*skr-5*			2.0	2.15E-28
K09D9.1				2.0	3.55E-22
					
**Down-regulated genes**					
ZK816.5	*dhs-26*	1.5	1.16E-10	2.3	3.77E-11
F58B3.3	*lys-6*			2.4	1.35E-17
F58B3.1	*lys-4*			2.3	3.80E-18
F58B3.2	*lys-5*			2.1	3.93E-18
Y48E1B.8				2.0	1.01E-15
Y39G10AR.6	*ugt-31*			2.0	5.16E-18

**Figure 3:**
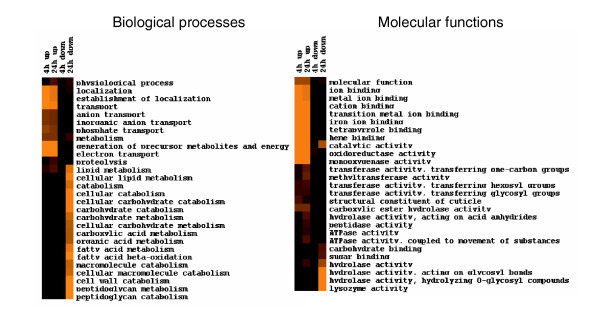
Biological processes and molecular functions enriched with cadmium-responsive genes. We used 286 genes that were significantly changed following a 24 h exposure to 100 μM cadmium and 86 genes that were significantly changed following a 4 h exposure in the GO analysis. GO terms with *p *< 0.05, and ≥4 changed genes in at least one of four conditions (up or down regulated after 4 or 24 h cadmium exposures) are displayed (Additional data files 3 and 4). The brighter the color, the more significant the enrichment of the pathway.

Microarray results were analyzed to identify the biological processes and molecular functions that are affected by cadmium exposure. To extend the scope of the analysis, 290 genes, whose expression levels were changed by cadmium by at least 1.5-fold (*p *< 0.001) following either 4 h or 24 h exposure, were used in the analysis (237 up-regulated and 53 down-regulated; Additional data file 2). Gene Ontology analysis indicated that *C. elegans *metabolic and localization pathways, which regulate establishment of localization and transportation of different chemical species (especially metal ions), were significantly enriched (*p *< 0.05) with up-regulated genes following the 4 h exposure (Figure 3, Additional data file 3). After a 24 h exposure, metabolic and localization pathways were enriched with both up- and down-regulated genes. Additional pathways that were overpopulated with down-regulated genes included fatty acid metabolism, cellular lipid metabolism and cell wall catabolism. Proteolysis pathways were enriched with up-regulated genes following a 24 h exposure, suggesting that increased protein degradation may occur after prolonged exposure to cadmium. The molecular functions enriched with over-expressed genes after both 4 h and 24 h exposures were catalytic activity and binding activities to many ion species, which agrees well with the results of the biological processes analysis (Figure 3, Additional data file 4).

Although GO analysis provides an overall understanding of the global transcription profile, current *C. elegans *GO data are not sufficient to predict the functions of all of the cadmium-responsive genes. Several *C. elegans *cadmium-regulated genes have been mapped to known metabolic pathways in the Kyoto Encyclopedia of Genes and Genomes (KEGG) database [18] (Table 2). Among these are four cytochrome P450 genes, which are involved in metabolism of both endogenous and exogenous compounds; gene W01A11.1, which is involved in degradation of tetrachloraethene; and *gst-38*, which is involved in phase II metabolism. The majority of the cadmium-responsive genes, however, are novel and have not been assigned GO categories or mapped to biochemical pathways. Of the 290 genes whose expression significantly changed following a 24 h cadmium exposure, only 83 (29%) have been assigned biological process GO terms. Similarly, only 109 (38%) of the genes following a 24 h exposure have been assigned molecular function GO terms (Additional data file 1).

**Table 2:** KEGG pathways for cadmium-responsive genes

Gene name	Description	KEGG pathway
T01B9.1	cyp-13A4*	Ascorbate and aldarate metabolism
T01B9.2	cyp-13A5*	Stilbene, coumarine and lignin biosynthesis
T01B9.3	ccp-13A6*	Gamma-hexachlorocycloheane degradation
T01B9.10	cyp-13A7*	Limonene and ponene degradation Fluorene degradation
		
W01A11.1	Predicted hydrolases or acyltransferases	Tetrachloraethene degradation
		
F35E8.8	*gst-38*	Glutathione metabolism

*Each of the *cyp *genes is found in each of the KEGG pathways listed in the right column.

### Functional analysis of cadmium-responsive genes using RNA interference

Of the 53 down-regulated genes (≥1.5-fold), 8 have previously reported RNAi phenotypes, including embryonic lethality, slow growth, larval growth arrest, and sterility (Table 3). This suggests that the suppression of the expression of these genes by cadmium may adversely effect embryonic development, growth or reproduction. Several of the up-regulated genes also have previously reported phenotypes, such as F57B9.3 (embryonic lethal, larval arrest) and *cyp-13A4 *(locomotion abnormal, slow growth) [19]. The biological consequence of changes in expression of the majority of the genes, and their roles in the defense against cadmium toxicity, however, are unknown. To investigate the relationship between cadmium-induced gene expression and resistance to cadmium toxicity, the effects of inhibiting the expression of the cadmium-responsive genes in the presence or absence of cadmium on *C. elegans *growth were determined.

**Table 3:** Published RNAi phenotypes of down-regulated genes

Target gene name	Description	24 h fold change	RNAi phenotype*	Reference
F09F7.4	Enoyl-CoA hydratase	1.8	Emb	[61]
			Gro	[19]
F22A3.6	Unknown	1.8	Emb	[62]
T15B7.1	Ficolin and related extracellular proteins	1.7	Emb	[63]
F52B11.4	Collagen (*col-133*)	1.7	Emb, Gro, Rup	[19]
R11G11.14	Triglyceride lipase-cholesterol esterase	1.6	Him	[64]
C55B7.4	Acyl CoA dehydrogenase (*acdh-1*)	1.5	Age	[65]
C25G4.6	Unknown	1.5	Ste	[66]
			Lva, Pvl, Stp	[67]
T04A8.5	Glutamine phosphoribosylpyrophosphate amidotransferase	1.5	Larval lethal-early (L1/L2), WT	[61]
			Lva	[63]

*The phenotypes are: Age, ageing alteration; Emb, abnormal embroygenesis; Gro, abnormal growth rate; Him, high incidence of males; Lva, larval arrest; Pvl, protruding vulva; Rup, exploded through vulva; Ste, sterile; Stp, sterile progeny; WT, wild type.

The expression of 92 cadmium-responsive genes, which were induced by cadmium (≥1.5-fold), was inhibited by RNAi in the presence of four different cadmium concentrations in an *mtl-2 *null background. In RNAi control animals, slow growth and uncoordinated movement were observed after cadmium exposure. Morphological changes (protruding vulva, multivulva) were occasionally observed at higher cadmium concentrations (100 and 200 μM). Lethality was not observed under any experimental condition. RNAi-mediated inhibition of 50 of the 92 genes tested resulted in slower growth in the presence of cadmium, compared to the RNAi control in the same treatment group (visual observation under microscope; Additional data file 5). The only gene that exhibited a morphological phenotype when inhibited by RNAi in the absence of cadmium was F57B9.3, which encodes a translation initiation related protein. As described previously [19,20], inhibition of F57B9.3 caused embryonic lethality and L1 larval arrest.

**Figure 4:**
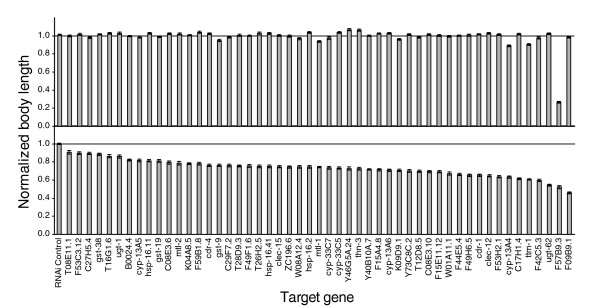
Effect of gene inhibition on *C. elegans *growth. The expression of target genes was inhibited using RNAi in the absence (upper panel) and presence (lower panel) of 100 μM cadmium. *mtl-2 *(gk125) mutant nematodes were grown on test plates for three days before collection (RNAi of gene *mtl-2 *was conducted using an *mtl-1 *null strain, *mtl-1 *(tm1770)). *C. elegans *body length, a measure of growth/development, was normalized to the mean body length in the RNAi control group under identical cadmium exposure conditions. Results are displayed as mean normalized body length ± SE (*n *= 200-500 nematodes).

To confirm and quantify the effect of the 50 cadmium-responsive genes that affected nematode growth in the presence of cadmium, we repeated the RNAi-mediated inhibition of these genes in the presence of 100 μM cadmium and measured nematode body length (as a measure of growth/development) using the COPAS Biosort [21]. Inhibiting the expression of these genes resulted in different degrees of slow growth in the presence of cadmium, compared to the RNAi control in the same cadmium treatment group (Figure 4, Additional data file 6). Based on the changes in cadmium sensitivity caused by RNAi, the genes were grouped into three classes, strong, medium and weak protective effects against cadmium toxicity (Additional data file 6). Several of the genes in the strong and medium category (*cdr-1*, *ttm-1*, *mtl-2*, and *mtl-1*) have been previously reported to be involved in cadmium detoxification [17,22,23]. However, the majority of the genes have not been shown to be involved in resistance to metal toxicity. GO molecular function analysis indicated that many of these genes have metal ion binding and catalytic activities (Table 4).

**Table 4:** Gene Ontology molecular functions of genes related to cadmium sensitivity

GO molecular function	Cadmium sensitivity genes
Iron ion binding	T10B9.1 F41B5.2 F41B5.3 T10B9.3 F49H6.5
Calcium ion binding	T08G5.10
Zinc ion binding	T26H2.5
Sugar binding	T27F6.2 Y73C8C.2
Monooxygenase activity	T10B9.1 F41B5.2 F41B5.3 T10B9.3
Transferase activity, transferring hexosyl groups	M88.1
Transferase activity, transferring acyl groups	F09B9.1
Methyltransferase activity	Y40B10A.7
Carboxylic ester hydrolase activity	K04A8.5 T08G5.10
Epoxide hydrolase activity	W01A11.1

The genes that had the strongest protective effects against cadmium toxicity in the growth assay were also examined for an effect on *C. elegans *reproduction. RNAi of *cyp-13A4 *or *thn-1 *resulted in a significant decrease in the number of *C. elegans *offspring when nematodes were exposed to cadmium, compared to the RNAi control in the same cadmium treatment group. RNAi did not significantly affect reproduction in the remainder of the tested genes (Table 5).

**Table 5:** Effects of RNAi and cadmium on *C. elegans *reproduction

Target gene	CGC gene name	Reproduction rate (minus cadmium)*	Reproduction rate (plus cadmium)*	Diff. of medians	*P *value^†^
T10B9.1	*cyp-13A4*	0.92, 0.92, 0.88, 0.96, 0.87	0.57, 0.59, 0.61, 0.79, 0.72	0.31	0.008
F28D1.3	*thn-1*	1.04, 1.02, 1.14, 1.03	0.89, 0.86, 0.92, 0.92	0.13	0.029
F35E8.11	*cdr-1*	1.01, 1.02, 0.94, 0.95, 1.19, 1.04	1.38, 1.21, 0.88, 0.79, 0.80, 0.84	0.15	0.394
Y39B6A.24		1.10, 1.03, 1.09	1.01, 1.21, 1.03	0.06	0.700
T26H2.5		0.88, 0.99, 0.81	0.74, 0.95, 1.00	-0.07	1.000
K11G9.6	*mtl-1*	0.85, 1.13, 0.99, 0.90, 1.07	0.97, 1.16, 1.12, 1.08, 1.12	-0.13	0.222
T27F6.2	*clec-12*	1.10, 0.90, 1.00, 1.03, 0.64, 0.90	1.05, 1.32, 1.42, 0.94, 1.32, 0.83	-0.24	0.180
C17H1.4		0.87, 0.89, 1.13	0.91, 1.06, 1.75	-0.15	0.686
C08E3.10		0.94, 0.93, 1.03	1.39, 1.24, 1.26	-0.32	0.100
M88.1	*ugt-62*	0.93, 1.03, 0.99, 1.05, 0.98, 0.89	1.08, 0.94, 1.34, 0.83, 0.95, 0.88	0.04	0.818
Y39E4A.2	*ttm-1*	0.73, 0.88, 1.05, 0.91, 0.94, 0.83, 1.01, 1.03	0.86, 0.82, 1.12, 0.91, 0.94, 0.93, 0.83, 0.85	0.04	0.721
F42C5.3		1.01, 0.94, 1.02, 1.12, 1.05, 0.99	1.06, 1.19, 1.10, 0.94, 0.91, 0.90	0.02	0.818
F53H2.1		1.06, 0.94, 1.00, 1.20, 0.95, 0.93	1.27, 1.30, 1.05, 1.05, 1.07, 0.96	-0.08	0.132
F09B9.1		0.76, 1.04, 1.06, 0.89, 1.13, 0.96, 1.01	1.04, 0.73, 0.95, 0.72, 0.88, 0.85, 0.92	0.13	0.073
Y46G5A.24		1.10, 0.94, 1.12	0.98, 0.87, 0.80	0.23	0.200

*The reproduction rate was calculated by comparing the number of offspring with the targeted gene knocked-down by RNAi to those without RNAi in the same cadmium-treatment group during a two day reproduction period after nematodes reached L4 stage of development. ^†^Wilcoxon Rank-sum tests were applied for significance tests.

### Protein interaction analysis reveals a novel pathway involved in the response to cadmium

**Figure 5:**
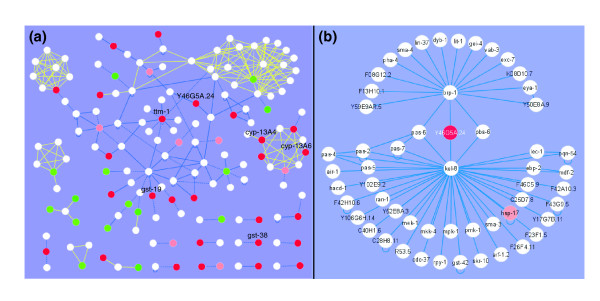
Protein interaction analysis using Cytoscape. High confidence interactions from yeast two-hybrid screens and the literature are displayed with solid blue lines; low confidence interactions from yeast two-hybrid screens are displayed with dashed blue lines; and predicted interactions are displayed with yellow lines. **(a) **Local networks involved in cadmium sensitivity. The network was filtered by showing nodes that were significantly up-regulated (red) or down-regulated (green) following a 24 h exposure to cadmium (≥1.5 fold, *p *≤ 0.001) and their first neighbors (white). The identified genes are those that affect cadmium sensitivity in the RNAi experiments. **(b) **The BRP-1-Y46G5A.24-KEL-8 interaction network. Immediate and secondary neighbors of Y46G5A.24 are displayed.

In order to further define the molecular mechanisms of the cadmium defense response, the program Cytoscape was used in protein interaction analysis to identify potential regulatory pathways [24]. *C. elegans *has a relatively small interaction database (approximately 3,000 proteins and approximately 5,000 interactions) [25]. A larger data set of predicted interactions in *C. elegans*, based on data from *Drosophila *and *Saccharomyces *interlogs, was recently released [26]. We merged the two data sets into an interaction network designated WI_combined. Of the 290 cadmium-responsive genes, 49 were mapped to the interaction network, including 6 genes that were functionally important in cadmium defense response, as identified in the RNAi analysis (Figure 5a). Among these functional local networks, Y46G5A.24, which encodes a β,β-carotene 15,15'-dioxygenase like protein, was highly cadmium-inducible and inhibition of this gene by RNAi resulted in hypersensitivity to cadmium (Table 1, Figure 4). Two proteins that interact with Y46G5A.24, KEL-8 and BRP-1, are themselves centers of other interactions.

**Figure 6:**
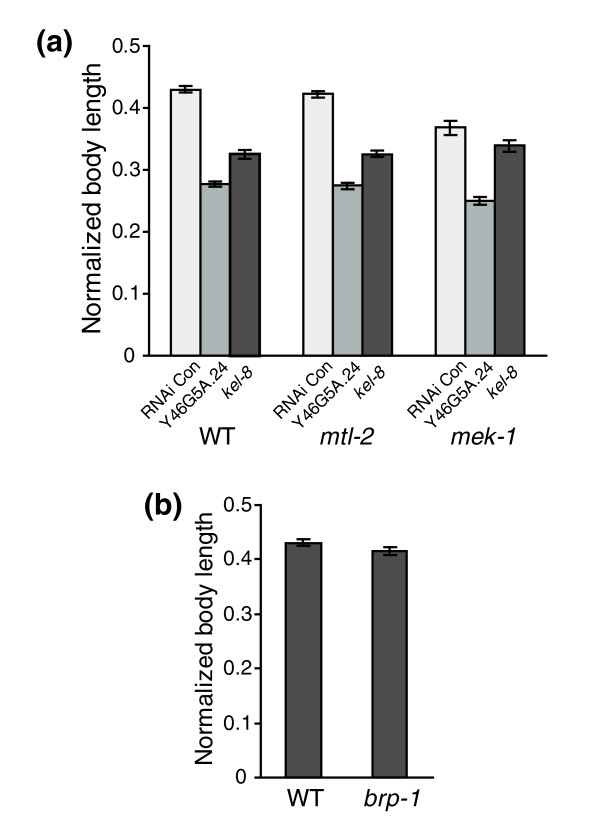
Functional analysis of the BRP-1-Y46G5A.24-KEL-8 interaction network. RNAi was performed in triplicate in the presence or absence of 100 μM cadmium. **(a) **The effect of inhibiting *kel-8 *or Y46G5A.24 in wild-type (WT), *mtl-2 *null (*mtl-2 *(gk125)) or *mek-1 *null (*mek-1*(ks54)) *C. elegans *on nematode growth in the presence of 100 μM cadmium. **(b) **Effects of cadmium exposure on growth of *brp-1 *null *C. elegans *(*brp-1 *(ok1084)). *C. elegans *body length of the cadmium exposed population was normalized to the mean body length in the same RNAi treatment group not exposed to cadmium. Results are displayed as mean body length ± SE (*n *= 200-500).

KEL-8 can interact with several proteins, including MEK-1, PMK-1, MPK-1 and MKK-4, which are components of the mitogen-activated protein kinase (MAPK) pathway (Figure 5b). The MAPK pathway is involved in the *C. elegans *heavy metal response [27,28]. Inhibiting the expression of Y46G5A.24 or *kel-8 *by RNAi resulted in enhanced sensitivity to cadmium exposure in wild-type and *mtl-2 *mutant *C. elegans *(Figure 6a). This suggests that both Y46G5A.24 and *kel-8 *can protect *C. elegans *from cadmium toxicity. The *mek-1 *mutant alone was slightly more sensitive to cadmium than wild-type nematodes. However, inhibition of *kel-8 *in *mek-1 *null background did not cause hypersensitivity to cadmium compared to *mek-1 *mutant alone, suggesting that the protective function of *kel-8 *against cadmium toxicity depends on the normal function of *mek-1 *(Figure 6a).

We also tested the function of *brp-*1, the other gene that was shown to interact with Y46G5A.24 *brp-1 *mutant nematodes showed a similar response to cadmium as wild-type nematodes, implying that *brp-1 *is not involved in the response to cadmium (Figure 6b).

## Discussion

### Identification of cadmium-responsive genes in *C. elegans*

Microarray technology has been used to examine the effects of cadmium exposure in a variety of organisms [29-32]. Although cadmium-regulated gene expression has been documented, in *C. elegans *there is inadequate information regarding the genome response to this metal. In the present study, well known cadmium-responsive *C. elegans *genes, *mtl-1*, *mtl-2*, *cdr-1 *and several heat shock protein genes, were identified, confirming the efficacy of the study. Previously, 49 *C. elegans *cDNAs, whose steady-state levels of expression change 2-6-fold in response to 24 h cadmium exposure, were identified using differential display [33]. Among these were *mtl-1*, *cdr-1*, *hsp-70*, and genes encoding collagen and metabolic proteins. Novillo *et al*. [34] also reported over-expression of *C. elegans cdr-1*, *mtl-2 *and collagen genes, as well as changes in the expression of metabolic genes, following a seven day exposure to cadmium. These expression data are similar to the present study, although some of their genes were not identified in the present analysis. This can be attributed to the difference in cadmium concentrations or exposure times, and methods of analysis. Another study conducted by Huffman *et al*. [22] also tested the *C. elegans *genome response following a 3 h exposure to 1 mM cadmium. However, the results are not comparable to our study because their study was conducted using a mutant strain, *glp-4(bn2)*, and only three replicate microarrays were performed.

Several gene families that have not been well-characterized in regard to cadmium exposure were identified. Among these are genes that encode phase I and phase II detoxifying proteins, innate immunity proteins, and ABC transporters. Cadmium exposure caused over-expression of fourteen P450 genes, six GST genes and five UDP-glucuronosyltransferase (UGT) genes. Cadmium also caused the down-regulation of one UGT gene. The P450 genes showed the most substantial expression changes, with changes between 1.5- to 32-fold, and many of them responded after a 4 h exposure. The cadmium-induced increase in P450 gene expression is similar to previous observations in *C. elegans *[35] and mammalian systems [36,37] but contrasts with the decreased expression observed in cadmium-exposed European flounder [30].

Cadmium affected the expression of several genes previously implicated in the nematode immune response. Four of the ten known lysozyme genes were down-regulated by cadmium, and four thaumatin/PR-5 family genes were up-regulated following cadmium exposure. *C. elegans *has 88 C-type lectins, a subset of which is inducible by infection and may function as a recognition tool in host defense [38,39]. The expression of eight *C. elegans *C-type lectin genes were affected by cadmium. There are several reports describing a relationship between cadmium exposure and changes in the immune response [40,41]. The immune response genes may be affected by cadmium due to the modulation of shared signal transduction pathways, such as the MAPK signaling cascade [27,42-44].

There are approximately 60 ABC transporter genes in the *C. elegans *genome. The expression of four of these genes, *pgp-1*, *pgp-8*, *pgp-9 *and *mrp-3*, was induced by cadmium, and *pmp-5 *was suppressed. A relationship between ABC transporter expression and cadmium exposure has also been observed in several species [45,46].

In addition to these known gene families, exposure to cadmium affected the expression of nuclear receptors (*nhr-206*, *mxl-3 *and *grl-23*), translation initiation factor (F57B9.3), *ins-7 *(insulin/IGF-1-like peptide), and genes with unknown functions. Interestingly, inhibition of many novel genes by RNAi resulted in hypersensitivity to cadmium, suggesting these genes have important roles in resistance to metal/cadmium toxicity.

GO analysis determined that the *C. elegans *genes that are over-expressed following a 4 h exposure to cadmium encode cellular trafficking proteins (localization/binding and transport) and metabolic enzymes. This suggests that the first response to cadmium intoxication is a transcriptional adjustment to maintain ion homeostasis and readjust the perturbed energy supply. Following a prolonged exposure (24 h), the proteolysis category was significantly enriched with over-expressed genes, suggesting an accumulation of damaged proteins. Cadmium exposure is associated with protein damage caused by metal binding to sulfhydryl groups or oxidative stress [47]. Cellular trafficking, fatty acid metabolism and cell wall metabolism categories were also enriched with down-regulated genes following a 24 h exposure to cadmium, indicating multiple cellular functions may be disrupted by cadmium toxicity.

### Discovery of novel genes and pathways involved in cadmium resistance

In *C. elegans*, *mtl-1*, *mtl-2*, *cdr-1 *and *ttm-1 *(Toxin-regulated target of p38 MAPK) are cadmium-responsive genes that function in resistance to cadmium toxicity [22]. *pcs-1*, a phytochelatin synthase, and *hmt-1*, an ATP-dependent phytochelatin transporter, were also able to protect *C. elegans *from cadmium toxicity [48-50]. However, the relationships between increased levels of transcription and biological function of most of the other *C. elegans *cadmium-responsive genes are unknown. To examine the function of the transcriptional change in response to cadmium, we combined functional genomics with microarray studies, and examined 92 cadmium-responsive genes in the presence and absence of cadmium. With one exception, inhibition of the expression of these genes did not affect *C. elegans *growth in the absence of metal. This suggests that most of the genes affected by cadmium are non-essential. Inhibition of these genes in the presence of metal resulted in hypersensitivity to cadmium, suggesting that these genes play important roles in the defense against cadmium toxicity. None of the tested genes showed lethal effects when inhibited in the presence of cadmium under the current experimental conditions. There are a couple of possible reasons: first, gene knockdown using RNAi is not 100% efficient and residual gene expression may be sufficient for defense against cadmium toxicity; and second, functional redundancy within the *C. elegans *genome could prevent lethal effects when the expression of only one of the redundant genes is affected.

By integrating the RNAi assay results into the protein interaction network, a novel signal pathway involved in cadmium resistance was discovered. The center of the network is Y46G5A.24, which encodes a β,β-carotene 15,15'-dioxygenase like protein. This protein shares 95.8% sequence identity with human β,β-carotene 15,15'-monooxygenase, an enzyme involved in the biosynthesis of retinoic acid. Cadmium has been shown to act synergistically with retinoic acid in the induction of limb-bud malformation in mice [51]. The Y46G5A.24 network includes *kel-8*, which encodes a signaling molecule containing a kelch-repeat, and *mek-1*, which is a major component in the MAPK signaling pathway [27,52]. RNAi results indicate that *kel-8 *is involved in protecting *C. elegans *from cadmium toxicity, and that the protective effect of *kel-8 *depends on the normal function of *mek-1*. Because *kel-8 *and *mek-1 *are both evolutionarily conserved, they may be components of a conserved metal-responsive signal transduction pathway.

Although only one local interaction network was examined in detail, there are several other local networks: *ttm-1*, *gst-9*, *cyp-13A4*, *cyp-12A6 *and *gst-38*. Further study of these functional local networks may provide additional information on the mechanisms involved in metal detoxification/resistance.

Many studies have demonstrated that large gene sets are induced in response to various stressors/toxicants. In general, these studies have been used to identify particular genes involved in the detoxification process. However, it has remained unclear if the global response to toxicant exposure is specific to detoxification of that stressor or a more general universal response. For example, cadmium exposure induces MAPK pathways, which affect the expression of genes that detoxify related stressors (metals, reactive oxygen species, and organic chemicals) but that do not defend against cadmium simply because these stressors affect common pathways. Alternatively, a response could be specific and largely only cadmium detoxification genes are over-expressed. By using RNAi to examine the role of 92 cadmium responsive genes in the resistance to cadmium toxicity, we find that 50 of these genes have at least some effect on nematode health when cadmium is present but not when it is absent. Moreover, because RNAi may only knock down gene function and not eliminate it entirely, it is plausible that even more of these genes could play a role in the resistance to cadmium toxicity. The fact that all the resistance genes identified in our initial visual screen had confirmed phenotypes in our secondary quantitative assay is indicative of the sensitivity of our system, allowing us to potentially identify resistance genes that play only a minor role in the response. Thus, in the *C. elegans *response to cadmium, approximately 21% of the 237 cadmium-inducible genes (≤1.5-fold) are involved in the resistance to cadmium toxicity (although these genes certainly could also be involved in detoxification of other stressors). These cadmium resistance genes include previously known genes involved in the cadmium response as well as several novel genes and pathways involved in cadmium detoxification.

## Conclusion

The results of the microarray and RNAi studies in *C. elegans *will help in the understanding of genomic responses to metals in higher organisms. Although cadmium-regulated expression of individual genes has been intensively studied, the biological consequences of global transcriptional changes caused by this metal were unexplored. In mammals, metallothioneins are the only cadmium-responsive proteins known to function in the cellular resistance to cadmium toxicity [16]. In the present study, we identified new cadmium-responsive genes in *C. elegans *that can protect nematodes from cadmium toxicity. The discovery of these novel genes involved in the resistance to cadmium toxicity provides valuable information in understanding the biological function of the transcriptional change caused by cadmium. Because more than 60% of *C. elegans *genes and many signaling pathways are evolutionarily conserved, these results contribute to understanding of functional roles of various genes in cadmium related diseases in humans.

## Materials and methods

### *C. elegans *strains

The following strains were used in this study: N2 Bristol; *mtl-2 *null, *mtl-2 *(gk125); *mtl-1 *null, *mtl-1 *(tm1770); *mek-1 *null, *mek-1 *(ks54); and *brp-1 *null, *brp-1 *(ok1084).

### Growth and collection of *C. elegans*

The Bristol N2 strain of *C. elegans *was cultured in S-medium with *Escherichia coli *OP50 as a food source, as previously described [33]. Nematodes were grown at 20°C and additional bacteria were added after three days to maintain an adequate food supply. For cadmium exposure studies, 250 ml of the *C. elegans *culture was placed into 1 L flasks. Nematodes were then exposed to cadmium under the following conditions prior to isolation: 24 h exposure at 0, 50, 100, or 200 μM cadmium; or 100 μM cadmium for 0, 4, 12, 24 or 36 h. Cadmium chloride, at the indicated final concentrations, was directly added to the culture medium. At the indicated times, nematodes were collected, washed, then rapidly frozen as pellets in liquid nitrogen and stored at -80°C, as previously described [33]. All exposure studies were preformed in triplicate.

### Isolation of total RNA and qRT-PCR

To isolate total RNA, frozen nematode pellets were ground into fine powder using a liquid nitrogen-chilled mortar and pestle before homogenization in TRIzol (Invitrogen, Carlsbad, CA, USA), as previously described [33]. Total RNA was subsequently purified using RNAeasy kits (Qiagen, Valencia, CA, USA) prior to qRT-PCR or microarray experiments.

The sequences of the oligonucleotide primers used in the qRT-PCR (Additional data file 7) were designed using Web Primer [53]. qRT-PCR was performed using QuantiTect SYBR Green RT-PCR kits (Qiagen) following the manufacture's instructions in an ABI Prism 7000 system (Applied Biosystems, Foster City, CA, USA). Three biological replicates for each treatment were prepared, and each biological replicate was measured three times.

### Microarray experiments and design

Total RNA was prepared from non-treated control nematodes and those exposed to 100 μM cadmium for 4 h and 24 h. For each condition, RNA samples were prepared from three independent cultures of *C. elegans*, and the RNA was isolated in triplicate. This generated nine replicates per treatment. RNA samples from cadmium-treated and control *C. elegans *were labeled using Low RNA Input Fluorescent Linear Amplification kits following the manufacture's protocols (Agilent Technologies, Inc, Santa Clara, CA, USA). Labeled cRNAs were hybridized to Agilent *C. elegans *oligonucleotide microarrays. These microarrays contained target probes for the entire *C. elegans *genome, approximately 20,000 open reading frames (Agilent Technologies). Dye-flips were performed for each pair of hybridizations, resulting in a total of 36 hybridizations. Data were extracted from the microarrays using Agilent's DNA microarray scanner and Feature Extraction software (Agilent Technologies). The data presented in this publication have been deposited in NCBIs Gene Expression Omnibus [54] and are accessible through GEO Series accession number GSE7535.

### Analysis of microarray expression data

GeneSpring GX (Agilent Technologies) was used for the initial analysis of expression data. Dye-flips were transformed and Lowess normalization was applied before further data processing. Genes with both red and green signals less than twice the background signal on more than two-thirds of the same treatment were excluded. Expression changes are described by fold change (expression ratio between treated and control signals). A cross gene error model was applied in the significance tests [55].

### Gene Ontology analysis of cadmium-responsive genes

The GO for 290 significantly changed genes (286 changed following the 24 h cadmium exposure and 86 changed following the 4 h exposure, fold change ≥1.5, *p *< 0.001) was assigned using GoMiner [56]. GO terms that met the two following criteria in at least one of four conditions are presented. The first criterion was that four or more genes were significantly changed in the GO term, and the second was that the *p *value of the enrichment was less than 0.05. The four conditions were: genes that are over-expressed after a 24 h exposure; genes that are under-expressed after a 24 h exposure; genes that are over-expressed after a 4 h exposure; and genes that are under-expressed after a 4 h exposure (Additional data files 3 and 4). Cluster [57] and TreeView [58] were used for the clustering and visualization of the results. *P *values of the GO terms were transformed into a positive number (-ln(*p *value)) to better visualize the data.

### Functional analysis of 92 cadmium-responsive genes using RNA interference: initial screen

RNAi was performed using the Ahringer bacterial RNAi library (MRC Gene Service, University of Cambridge, UK). To increase the sensitivity of the RNAi screen, a nematode strain carrying a deletion in the *C. elegans *metallothionein-2 gene (*mtl-2(gk125) V*) was used. This strain was backcrossed four times to wild-type nematodes prior to use. This mutation did not affect the growth or reproduction of nematodes under experimental conditions, but did increase the sensitivity of *C. elegans *to cadmium when *mtl-1 *was inhibited by RNAi. The feeding protocol was as previously described, with the following modifications [59]. Synchronized L1 larva were put on standard NGM plates with *E*. *coli *OP50 as food for approximately 31 h and allowed to develop into L4 larva. About 25 early L4 nematodes were then transferred to each well of a 6-well NGM plate, containing 1 mM IPTG (isopropyl-β-D-thiogalactopyranoside) and 25 μg/ml carbenicillin, seeded with different RNAi expressing bacteria. Plates were then incubated at 20°C for approximately 40 h. After the initial feeding period, 2 gravid adults from each well were then transferred to test plates containing RNAi bacteria and either 0, 50, 100, or 200 μM cadmium. Control RNAi (empty vector) exposures were also performed for each cadmium treatment. Adults were allowed to lay eggs for approximately 12 h before removal. F1 nematodes were then grown on test plates for three additional days at 20°C. The following phenotypes were scored every 24 h: lethality (larval lethality); development (slow growth, larval arrest); morphology (gross body morphology defect); and movement (uncoordinated, sluggish, and paralyzed).

The initial screen of 92 genes (whose expression increased following cadmium exposure; Additional data file 5) was performed in duplicate. Genes that showed increased sensitivity to cadmium after RNAi compared to the RNAi control in at least one replicate study were chosen for a second round of RNAi screening.

### Functional analysis of 50 cadmium-responsive genes using RNA interference: second screen

#### Quantification of effect of RNAi on *C. elegans *growth

Experiments were performed as described in the initial screen except only two conditions were tested, no cadmium or 100 μM cadmium. Each exposure was performed in triplicate. At the end of the 3 day growth period, F1 nematodes were washed off the 6-well plates using M9 buffer and transferred onto 96-well plates. Body size measurements, as represented by nematode body length (time of flight (TOF)) were measured using the COPAS Biosort [21].

#### Quantification of effect of RNAi on *C. elegans *reproduction

Synchronized L1s were transferred onto RNAi feeding plates. After approximately 40 h, 3 L4 nematodes were transferred to fresh plates containing RNAi expressing bacteria (0 or 100 μM cadmium). After two days, the F1 nematodes were counted using the COPAS Biosort. Only those plates that had all three adults at the end of the test were measured. Three to eight replicate plates were successfully counted for each RNAi treatment.

### Data analysis

For qRT-PCR assays, all measurements were normalized to *mlc-2*, and fold change of each gene was normalized to that observed in the non-treated *C. elegans *samples. Final results are presented by mean log_2_fold ± standard error (SE; *n *= 3).

In the study of RNAi effects on nematode growth, body length means for control nematodes differed slightly across replicate experiments. To account for differing body length means among animal cohorts, body length measurements on RNAi plates were normalized to the RNAi control by dividing by the mean body length of the RNAi control nematodes of the same cadmium treatment, unless another method is described. Then measurements from all replicates in the same cadmium and RNAi treatment were pooled together. To compare the normalized mean body length in the presence and absence of cadmium for the same RNAi treatment, Welch two-sample *t*-tests were performed because these tests accounted for differences in variance between groups [60]. Results were presented in terms of the difference in the normalized mean body length in the presence and absence of cadmium (*n *= ~200-500).

In the RNAi tests of *C. elegans *reproduction, the reproduction rate under RNAi was calculated by dividing the number of progeny on the RNAi plate by that of the RNAi control in the same cadmium treatment. Because reproduction rates calculated from replicates were not normally distributed, Wilcoxon Rank-sum tests were performed to assess significant differences in the reproduction rates with or without cadmium.

## Additional data files

The following additional data are available with the online version of this paper. Additional data file 1 is a figure showing the cadmium-responsive genes that have been mapped to biological processes and molecular functions following 4 and 24 h cadmium exposures. Additional data file 2 is a table listing genes that were up- or down-regulated (≥1.5 fold, *p *< 0.001) following 4 h and 24 h cadmium exposures. Additional data file 3 is a table displaying significantly enriched biological processes following 4 h and 24 h cadmium exposures and genes in the pathway that are cadmium-responsive. Additional data file 4 is a table displaying significantly enriched molecular functions following 4 h and 24 h cadmium exposures and genes in the pathway that are cadmium-responsive. Additional data file 5 is a table listing the cadmium-responsive genes tested in the first round RNAi screen. Additional data file 6 is a table summarizing the effect of RNAi and cadmium on *C. elegans *body size in the second round RNAi screen using COPAS BioSort. Additional data file 7 is a table listing the primer sequences used in qRT-PCR.

## Supplementary Material

Additional data file 1 Cadmium-responsive genes that have been mapped to biological processes and molecular functions following 4 and 24 h cadmium exposures. Click here for file

Additional data file 2 Genes up- or down-regulated (≥1.5 fold, *p *< 0.001) following 4 h and 24 h cadmium exposures. Click here for file

Additional data file 3 Significantly enriched biological processes following 4 h and 24 h cadmium exposures and genes in the pathway that are cadmium-responsive. Click here for file

Additional data file 4 Significantly enriched molecular functions following 4 h and 24 h cadmium exposures and genes in the pathway that are cadmium-responsive. Click here for file

Additional data file 5 Cadmium-responsive genes tested in the first round RNAi screen. Click here for file

Additional data file 6 Effect of RNAi and cadmium on *C. elegans *body size in the second round RNAi screen using COPAS BioSort. Click here for file

Additional data file 7 Primer sequences used in qRT-PCR. Click here for file
