# Supplementary material for: Toxicogenomic analysis of Caenorhabditis elegans reveals novel genes and pathways involved in the resistance to cadmium toxicity
Source: Genome Biol. 2007 Jun 25;8(6):R122. doi: 10.1186/gb-2007-8-6-r122 (PMC2394766; doi:10.1186/gb-2007-8-6-r122)
Supplement: Additional data file 2 — Genes up- or down-regulated (≥1.5 fold, p < 0.001) following 4 h and 24 h cadmium exposures. [file gb-2007-8-6-r122-S2.doc]

| **Gene Name** | **CGC**  **Gene Name** | **Regulation** | **4 h Exposure** | | **24 h Exposure** | |
| --- | --- | --- | --- | --- | --- | --- |
|  |  |  | **Fold Change** | **p-value** | **Fold Change** | **p-value** |
| F35E8.11 | *cdr-1* | up | 73.4 | 1.00E-43 | 111.4 | 3.10E-43 |
| R04D3.1 | *cyp-14A4* | up | 14.9 | 5.32E-37 | 32.4 | 4.02E-34 |
| T08G5.10 | *mtl-2* | up | 28.7 | 1.70E-38 | 31.7 | 0 |
| Y46G5A.24 |  | up | 7.4 | 1.32E-37 | 18.3 | 6.16E-37 |
| T26H2.5 |  | up | 10.1 | 6.57E-27 | 15.2 | 2.88E-38 |
| K11G9.6 | *mtl-1* | up | 17.1 | 6.28E-40 | 15.0 | 4.71E-39 |
| F28D1.4 | *thn-3* | up | 3.6 | 2.69E-23 | 14.3 | 6.80E-33 |
| F56A4.5 |  | up | 6.5 | 1.96E-29 | 11.8 | 1.32E-32 |
| Y39B6A.24 |  | up | 3.4 | 7.06E-31 | 11.4 | 4.48E-42 |
| F28D1.3 | *thn-1* | up | 4.0 | 7.88E-34 | 9.9 | 1.50E-42 |
| Y59E9AR.4 | *thn-5* | up | 3.7 | 2.50E-35 | 8.5 | 4.87E-42 |
| AC3.7 | *ugt-1* | up | 5.4 | 4.16E-42 | 8.2 | 3.46E-37 |
| C02A12.1 | *gst-33* | up | 3.8 | 2.57E-33 | 7.8 | 2.15E-33 |
| T10B9.1 | *cyp-13A4* | up | 4.3 | 6.38E-40 | 7.1 | 1.12E-35 |
| F08F8.5 |  | up |  |  | 7.1 | 1.06E-28 |
| F41B5.2 | *cyp-33C7* | up | 5.3 | 6.86E-30 | 6.9 | 5.18E-42 |
| T10B9.10 | *cyp-13A7* | up | 6.3 | 2.57E-26 | 6.9 | 4.00E-26 |
| T08G5.1 |  | up | 4.9 | 1.20E-27 | 6.8 | 4.04E-32 |
| T16G1.6 |  | up | 4.5 | 1.16E-41 | 6.1 | 3.91E-36 |
| F53C3.12 |  | up | 3.9 | 1.07E-31 | 5.7 | 2.12E-40 |
| B0507.8 |  | up | 1.8 | 4.14E-09 | 5.6 | 2.51E-32 |
| C08E3.6 |  | up |  |  | 5.2 | 2.02E-27 |
| F35E8.8 | *gst-38* | up | 1.6 | 3.05E-20 | 5.0 | 1.41E-28 |
| T10B9.2 | *cyp-13A5* | up | 2.9 | 3.11E-36 | 4.4 | 1.22E-34 |
| T18D3.3 |  | up | 3.3 | 2.18E-31 | 4.3 | 1.45E-35 |
| C31B8.4 |  | up | 1.8 | 5.34E-09 | 4.3 | 2.57E-37 |
| C27H5.4 |  | up | 2.4 | 1.90E-27 | 4.1 | 1.52E-32 |
| C17H1.8 |  | up |  |  | 4.0 | 5.83E-19 |
| C17H1.3 |  | up | 2.7 | 5.00E-17 | 4.0 | 7.58E-25 |
| Y40B10A.6 |  | up | 2.6 | 2.99E-20 | 3.8 | 6.72E-23 |
| F28D1.5 | *thn-2* | up | 3.4 | 3.45E-30 | 3.7 | 3.51E-35 |
| T01C3.4 |  | up | 1.6 | 3.18E-07 | 3.7 | 3.88E-32 |
| Y40B10A.7 |  | up | 2.4 | 1.41E-20 | 3.5 | 1.59E-22 |
| F49F1.6 |  | up | 2.4 | 6.64E-33 | 3.4 | 2.19E-38 |
| F15E11.12 |  | up |  |  | 3.4 | 1.88E-10 |
| W08A12.4 |  | up | 1.6 | 2.66E-12 | 3.3 | 2.54E-27 |
| F45D11.4 |  | up | 2.3 | 1.52E-22 | 3.3 | 6.57E-31 |
| F48C1.9 |  | up |  |  | 3.3 | 2.97E-14 |
| R05D8.9 |  | up | 1.8 | 9.43E-18 | 3.2 | 4.16E-25 |
| K04A8.5 |  | up | 2.1 | 1.04E-25 | 3.0 | 2.50E-23 |
| ZC196.6 |  | up |  |  | 3.0 | 1.18E-26 |
| C08E3.10 |  | up | 1.6 | 2.44E-08 | 3.0 | 1.58E-30 |
| Y73C8C.2 |  | up | 1.8 | 1.45E-22 | 3.0 | 3.27E-28 |
| T08E11.1 |  | up |  |  | 2.9 | 5.78E-24 |
| C54D10.8 |  | up |  |  | 2.9 | 3.96E-24 |
| C17H1.4 |  | up |  |  | 2.9 | 5.74E-21 |
| Y39G8B.7 |  | up |  |  | 2.8 | 1.55E-26 |
| C45G7.3 |  | up |  |  | 2.8 | 7.31E-23 |
| F49F1.7 |  | up | 2.2 | 2.93E-20 | 2.8 | 6.66E-24 |
| F15B9.6 |  | up | 1.5 | 2.29E-17 | 2.7 | 4.03E-33 |
| F26F2.3 |  | up | 2.4 | 1.77E-14 | 2.7 | 1.66E-19 |
| F37B1.8 | *gst-19* | up | 2.0 | 8.18E-24 | 2.7 | 2.40E-27 |
| ZK742.3 |  | up | 1.5 | 9.27E-14 | 2.7 | 1.04E-24 |
| C17H1.9 |  | up |  |  | 2.7 | 8.10E-18 |
| E02A10.2 | *grl-23* | up | 2.1 | 2.47E-07 | 2.6 | 3.93E-19 |
| T07D10.4 | *clec-15* | up | 1.7 | 6.03E-10 | 2.6 | 1.51E-22 |
| F45D11.14 |  | up | 2.1 | 9.71E-09 | 2.6 | 2.43E-14 |
| C08E3.1 |  | up |  |  | 2.4 | 1.12E-19 |
| F37B1.1 | *gst-24* | up |  |  | 2.4 | 1.02E-18 |
| T10B9.3 | *cyp-13A6* | up | 1.9 | 2.08E-25 | 2.4 | 5.68E-25 |
| W01A11.1 |  | up | 2.1 | 2.01E-28 | 2.3 | 1.28E-31 |
| C47A10.1 | *pgp-9* | up | 1.8 | 1.09E-20 | 2.3 | 3.32E-21 |
| F42C5.3 |  | up | 1.6 | 1.37E-11 | 2.3 | 3.76E-20 |
| B0024.4 |  | up |  |  | 2.3 | 9.01E-17 |
| B0507.10 |  | up |  |  | 2.3 | 2.11E-23 |
| Y105C5A.12 |  | up |  |  | 2.3 | 1.06E-19 |
| T27E4.2 | *hsp-16.11* | up |  |  | 2.3 | 9.06E-30 |
| ZK643.8 | *grl-25* | up | 1.8 | 7.23E-07 | 2.2 | 2.62E-19 |
| F15E6.8 |  | up |  |  | 2.2 | 8.03E-23 |
| F13H6.3 |  | up | 1.7 | 3.31E-25 | 2.2 | 6.26E-31 |
| K02E2.7 |  | up |  |  | 2.2 | 1.25E-22 |
| F57B9.3 |  | up |  |  | 2.2 | 1.18E-19 |
| Y19D10B.7 |  | up |  |  | 2.2 | 2.44E-13 |
| T16G1.5 |  | up | 3.0 | 1.65E-30 | 2.2 | 1.02E-29 |
| F49H6.5 |  | up | 1.5 | 1.18E-09 | 2.2 | 1.19E-16 |
| C29F7.1 |  | up | 1.9 | 2.15E-24 | 2.2 | 3.55E-30 |
| F44E7.5 |  | up | 1.5 | 6.49E-21 | 2.2 | 3.59E-29 |
| M88.1 | *ugt-62* | up | 1.6 | 1.06E-23 | 2.2 | 2.78E-34 |
| F53H2.1 |  | up |  |  | 2.2 | 2.47E-23 |
| B0284.2 |  | up |  |  | 2.1 | 5.41E-26 |
| C54D10.7 |  | up |  |  | 2.1 | 2.83E-21 |
| F56C3.9 |  | up | 1.5 | 3.21E-13 | 2.1 | 4.20E-23 |
| T27F6.2 | *clec-12* | up |  |  | 2.1 | 1.45E-18 |
| D2023.7 | *col-158* | up | 1.8 | 6.26E-07 | 2.1 | 6.44E-15 |
| C29F7.2 |  | up |  |  | 2.1 | 1.41E-30 |
| T12D8.5 |  | up |  |  | 2.1 | 5.12E-22 |
| F41B5.3 | *cyp-33C5* | up | 1.6 | 8.21E-20 | 2.1 | 1.21E-24 |
| F15A4.8 |  | up |  |  | 2.0 | 2.08E-22 |
| T28D9.3 |  | up | 1.7 | 3.40E-24 | 2.0 | 7.68E-30 |
| F09B9.1 |  | up | 1.7 | 8.66E-20 | 2.0 | 1.63E-32 |
| Y75B8A.28 |  | up |  |  | 2.0 | 3.06E-20 |
| F15E11.1 |  | up |  |  | 2.0 | 3.41E-11 |
| B0284.4 |  | up |  |  | 2.0 | 4.57E-13 |
| F47H4.10 | *skr-5* | up |  |  | 2.0 | 2.15E-28 |
| K09D9.1 |  | up |  |  | 2.0 | 3.55E-22 |
| F15E11.15 |  | up |  |  | 1.9 | 9.01E-06 |
| C29F3.7 |  | up | 1.9 | 2.32E-27 | 1.9 | 9.08E-30 |
| C17H1.7 |  | up |  |  | 1.9 | 1.12E-26 |
| F59B1.8 |  | up | 1.7 | 2.26E-17 | 1.9 | 4.56E-24 |
| F44C8.1 | *cyp-33C4* | up | 1.6 | 2.07E-25 | 1.9 | 2.07E-29 |
| F43C11.8 |  | up |  |  | 1.9 | 5.63E-16 |
| W06G6.11 |  | up |  |  | 1.9 | 3.11E-13 |
| M01D1.2 | *math-34* | up |  |  | 1.9 | 7.03E-21 |
| C13A2.9 |  | up | 1.7 | 2.10E-06 | 1.9 | 2.85E-17 |
| F49F1.1 |  | up | 1.5 | 1.73E-12 | 1.8 | 1.28E-13 |
| F23B2.10 |  | up |  |  | 1.8 | 2.05E-11 |
| T27E7.6 |  | up |  |  | 1.8 | 1.04E-17 |
| ZC513.8 | *col-43* | up | 1.6 | 4.54E-07 | 1.8 | 5.73E-17 |
| T21E8.3 | *pgp-8* | up |  |  | 1.8 | 4.08E-12 |
| F53B2.8 |  | up |  |  | 1.8 | 4.34E-26 |
| R02D3.6 | *grl-19* | up | 1.7 | 4.23E-07 | 1.8 | 5.46E-18 |
| R12A1.4 | *ges-1* | up |  |  | 1.8 | 3.39E-31 |
| B0213.15 | *cyp-34A9* | up | 1.5 | 1.25E-14 | 1.8 | 1.52E-24 |
| B0294.1 |  | up |  |  | 1.8 | 6.44E-23 |
| M03F8.4 |  | up |  |  | 1.8 | 2.87E-21 |
| Y51A2B.1 |  | up |  |  | 1.8 | 1.67E-17 |
| F08G5.6 |  | up |  |  | 1.8 | 2.23E-20 |
| F10D2.11 | *ugt-41* | up |  |  | 1.7 | 1.15E-14 |
| T21C9.8 |  | up |  |  | 1.7 | 6.09E-23 |
| Y46H3A.3 | *hsp-16.2* | up |  |  | 1.7 | 2.36E-15 |
| C31A11.5 |  | up | 2.0 | 7.33E-24 | 1.7 | 3.35E-18 |
| F42A9.5 | *cyp-33E2* | up |  |  | 1.7 | 7.88E-23 |
| F15B9.1 | *far-3* | up |  |  | 1.7 | 6.09E-15 |
| T08B1.3 | *alh-5* | up |  |  | 1.7 | 3.29E-18 |
| F12B6.2 |  | up | 1.6 | 1.78E-19 | 1.7 | 1.99E-24 |
| K10B2.2 |  | up |  |  | 1.7 | 1.83E-24 |
| Y19D10A.9 |  | up |  |  | 1.7 | 2.32E-15 |
| T27E4.3 | *hsp-16.48* | up |  |  | 1.7 | 1.21E-19 |
| F46B6.8 |  | up |  |  | 1.7 | 3.61E-21 |
| F27D9.2 |  | up |  |  | 1.7 | 1.05E-16 |
| Y47H10A.3 |  | up |  |  | 1.7 | 4.27E-19 |
| ZK666.7 | *clec-61* | up |  |  | 1.7 | 5.97E-22 |
| F45D11.1 |  | up |  |  | 1.7 | 1.90E-15 |
| T26F2.2 |  | up |  |  | 1.7 | 1.67E-20 |
| K10B2.2 |  | up |  |  | 1.7 | 8.01E-21 |
| T07H3.2 | *bath-46* | up |  |  | 1.7 | 4.34E-15 |
| R05F9.5 | *gst-9* | up |  |  | 1.7 | 6.02E-15 |
| T10H4.12 | *cpr-3* | up |  |  | 1.7 | 2.56E-23 |
| F08H9.9 | *clec-55* | up |  |  | 1.7 | 2.47E-21 |
| C06E4.8 |  | up |  |  | 1.6 | 1.43E-20 |
| W09G12.7 |  | up |  |  | 1.6 | 2.26E-20 |
| C30F2.4 |  | up |  |  | 1.6 | 2.71E-18 |
| Y58A7A.5 |  | up |  |  | 1.6 | 3.28E-10 |
| K12D9.1 |  | up |  |  | 1.6 | 7.72E-09 |
| Y46G5A.20 |  | up |  |  | 1.6 | 2.79E-16 |
| F47H4.2 |  | up |  |  | 1.6 | 1.15E-18 |
| F26F2.4 |  | up |  |  | 1.6 | 7.78E-07 |
| C50F7.5 |  | up |  |  | 1.6 | 6.38E-14 |
| C12C8.1 | *hsp-70* | up | 1.5 | 1.49E-15 | 1.6 | 5.39E-24 |
| F02D10.1 | *col-183* | up |  |  | 1.6 | 2.01E-22 |
| F01D5.5 |  | up |  |  | 1.6 | 6.14E-17 |
| Y54G2A.8 | *clec-82* | up |  |  | 1.6 | 1.25E-16 |
| Y46H3A.2 | *hsp-16.41* | up |  |  | 1.6 | 1.46E-19 |
| C15C8.3 |  | up |  |  | 1.6 | 2.02E-15 |
| W09G10.1 | *col-72* | up |  |  | 1.6 | 4.19E-14 |
| F26F2.1 |  | up |  |  | 1.6 | 1.06E-12 |
| F41B5.7 | *cyp-33C6* | up |  |  | 1.6 | 1.00E-15 |
| C18A11.1 |  | up |  |  | 1.6 | 4.62E-19 |
| C10C5.2 |  | up |  |  | 1.6 | 3.06E-13 |
| ZC204.12 |  | up |  |  | 1.6 | 2.60E-17 |
| F35E12.8 |  | up |  |  | 1.6 | 4.24E-19 |
| T21D9.1 | *col-164* | up | 1.5 | 2.53E-06 | 1.6 | 4.25E-15 |
| F55H12.2 |  | up |  |  | 1.6 | 7.32E-17 |
| Y9C9A.1 |  | up |  |  | 1.6 | 2.96E-11 |
| K01A2.2 | *far-7* | up |  |  | 1.6 | 7.32E-17 |
| T07C12.9 |  | up |  |  | 1.6 | 1.59E-14 |
| F44E5.4 |  | up | 1.5 | 6.48E-12 | 1.6 | 2.24E-17 |
| C16C4.10 | *math-5* | up |  |  | 1.6 | 2.07E-14 |
| ZC204.13 |  | up |  |  | 1.6 | 4.15E-12 |
| Y43F8A.3 |  | up |  |  | 1.6 | 8.74E-19 |
| Y47H10A.5 |  | up |  |  | 1.6 | 3.99E-16 |
| T01D3.6 |  | up |  |  | 1.6 | 4.22E-18 |
| R07B7.13 | *nhr-206* | up |  |  | 1.6 | 6.06E-21 |
| Y39E4A.2 | *ttm-1* | up | 1.5 | 1.14E-12 | 1.6 | 6.17E-16 |
| F07G11.1 |  | up | 1.5 | 2.82E-06 | 1.6 | 3.31E-09 |
| F46B3.1 |  | up |  |  | 1.6 | 3.39E-06 |
| T28A11.19 |  | up |  |  | 1.6 | 1.52E-13 |
| F42G2.4 |  | up |  |  | 1.5 | 5.12E-15 |
| T01D3.6 |  | up |  |  | 1.5 | 2.34E-20 |
| F14F7.2 | *cyp-13A11* | up | 1.5 | 4.04E-13 | 1.5 | 1.01E-13 |
| F45D11.2 |  | up |  |  | 1.5 | 1.18E-21 |
| PDB1.1 |  | up |  |  | 1.5 | 6.26E-21 |
| F59D6.3 |  | up |  |  | 1.5 | 3.83E-14 |
| K01D12.11 | *cdr-4* | up |  |  | 1.5 | 2.37E-19 |
| C08E8.4 |  | up |  |  | 1.5 | 1.79E-13 |
| W03G1.5 |  | up |  |  | 1.5 | 6.85E-14 |
| F28F8.2 | *acs-2* | up |  |  | 1.5 | 1.60E-14 |
| VC5.3 | *npa-1* | up |  |  | 1.5 | 2.16E-14 |
| Y45F10D.6 |  | up |  |  | 1.5 | 2.09E-06 |
| C01B10.10 |  | up |  |  | 1.5 | 3.03E-22 |
| K01A2.2 | *far-7* | up |  |  | 1.5 | 1.53E-17 |
| M01G12.9 |  | up |  |  | 1.5 | 2.26E-16 |
| AC3.8 | *ugt-2* | up | 1.5 | 8.78E-18 | 1.5 | 4.15E-22 |
| F52E1.7 | *hsp-17* | up |  |  | 1.5 | 3.10E-18 |
| T24B8.5 |  | up |  |  | 1.5 | 5.04E-12 |
| T08A9.12 | *spp-2* | up |  |  | 1.5 | 9.43E-12 |
| F57G4.1 |  | up |  |  | 1.5 | 4.97E-16 |
| C08E3.7 |  | up |  |  | 1.5 | 1.94E-14 |
| ZC196.4 |  | up |  |  | 1.5 | 1.58E-16 |
| F55G11.4 |  | up |  |  | 1.5 | 8.74E-22 |
| VC5.3 | *npa-1* | up |  |  | 1.5 | 1.52E-12 |
| K08E7.9 | *pgp-1* | up |  |  | 1.5 | 1.56E-20 |
| F46B3.17 | *col-163* | up | 1.5 | 2.10E-07 | 1.5 | 1.46E-13 |
| T20H9.4 | *fbxa-73* | up |  |  | 1.5 | 2.18E-15 |
| H06H21.8 |  | up |  |  | 1.5 | 1.83E-18 |
| F37B1.4 | *gst-15* | up |  |  | 1.5 | 3.97E-18 |
| M05D6.7 | *gbh-2* | up |  |  | 1.5 | 2.33E-18 |
| F42A9.4 | *cyp-33E3* | up |  |  | 1.5 | 1.59E-19 |
| ZK455.4 | *asm-2* | up |  |  | 1.5 | 1.10E-20 |
| F22B7.9 |  | up |  |  | 1.5 | 3.35E-20 |
| C06E4.3 |  | up |  |  | 1.5 | 1.85E-13 |
| Y75B8A.27 | *pqn-92* | up |  |  | 1.5 | 1.75E-14 |
| W03D2.7 |  | up |  |  | 1.5 | 2.02E-12 |
| F36D3.9 | *cpr-2* | up |  |  | 1.5 | 2.22E-15 |
| M01G12.12 | *rrf-2* | up |  |  | 1.5 | 2.97E-17 |
| Y6E2A.5 |  | up |  |  | 1.5 | 1.37E-16 |
| C08F11.3 |  | up |  |  | 1.5 | 7.92E-11 |
| F11A5.15 |  | up |  |  | 1.5 | 1.21E-14 |
| F19C7.2 |  | up |  |  | 1.5 | 3.08E-20 |
| F54B8.4 |  | up |  |  | 1.5 | 2.55E-17 |
| ZK1251.2 | *ins-7* | up |  |  | 1.5 | 5.16E-16 |
| B0564.3 |  | up |  |  | 1.5 | 3.96E-09 |
| E03G2.2 | *mrp-3* | up |  |  | 1.5 | 3.87E-17 |
| K04F1.9 |  | up |  |  | 1.5 | 6.07E-19 |
| F19C7.4 |  | up |  |  | 1.5 | 2.10E-22 |
| ZC443.6 | *ugt-16* | up |  |  | 1.5 | 1.10E-21 |
| F02A9.1 |  | up |  |  | 1.5 | 4.88E-04 |
| F52E1.5 |  | up |  |  | 1.5 | 3.76E-15 |
| C25H3.10 |  | up |  |  | 1.5 | 2.26E-16 |
| C56A3.1 | *grl-17* | up | 1.5 | 3.79E-07 | 1.5 | 9.87E-14 |
| C32F10.4 |  | up |  |  | 1.5 | 3.10E-16 |
| F46G10.6 | *mxl-3* | up |  |  | 1.5 | 2.60E-11 |
| Y110A2AL.2 |  | up |  |  | 1.5 | 3.16E-14 |
| C45H4.17 | *cyp-33C2* | up | 1.8 | 2.56E-18 |  |  |
| Y37A1B.5 |  | up | 1.7 | 6.07E-23 |  |  |
| F49E12.10 |  | up | 1.5 | 2.99E-08 |  |  |
| F42G8.8 |  | up | 1.7 | 3.10E-21 |  |  |
| F58B3.3 | *lys-6* | down |  |  | 2.4 | 1.35E-17 |
| F58B3.1 | *lys-4* | down |  |  | 2.3 | 3.80E-18 |
| ZK816.5 | *dhs-26* | down | 1.5 | 1.16E-10 | 2.3 | 3.77E-11 |
| F58B3.2 | *lys-5* | down |  |  | 2.1 | 3.93E-18 |
| Y48E1B.8 |  | down |  |  | 2.0 | 1.01E-15 |
| Y39G10AR.6 | *ugt-31* | down |  |  | 2.0 | 5.16E-18 |
| Y4C6B.6 |  | down |  |  | 1.9 | 1.58E-16 |
| B0218.6 | *clec-51* | down |  |  | 1.9 | 8.00E-17 |
| F08A8.2 |  | down |  |  | 1.9 | 1.29E-12 |
| T09F5.9 | *clec-47* | down |  |  | 1.8 | 4.63E-11 |
| K09F5.2 | *vit-1* | down |  |  | 1.8 | 3.05E-13 |
| F09F7.4 |  | down |  |  | 1.8 | 2.62E-14 |
| T05E12.6 |  | down |  |  | 1.8 | 5.10E-16 |
| F17E9.11 | *lys-10* | down |  |  | 1.8 | 6.68E-14 |
| F22A3.6 |  | down |  |  | 1.8 | 2.63E-16 |
| F09F7.4 |  | down |  |  | 1.7 | 1.18E-11 |
| Y34F4.2 |  | down |  |  | 1.7 | 1.23E-12 |
| C17C3.12 | *acdh-2* | down |  |  | 1.7 | 8.05E-15 |
| C24B9.3 |  | down |  |  | 1.7 | 4.07E-16 |
| T15B7.1 |  | down |  |  | 1.7 | 1.14E-08 |
| F52B11.4 | *col-133* | down |  |  | 1.6 | 2.85E-11 |
| F44G3.2 |  | down |  |  | 1.6 | 2.18E-11 |
| Y38H6C.1 |  | down |  |  | 1.6 | 5.22E-11 |
| F49E12.9 |  | down |  |  | 1.6 | 1.46E-14 |
| F21C10.9 |  | down |  |  | 1.6 | 6.06E-15 |
| R11G11.14 |  | down |  |  | 1.6 | 6.62E-14 |
| Y46C8AL.3 | *clec-70* | down |  |  | 1.6 | 1.91E-14 |
| R05F9.12 |  | down |  |  | 1.6 | 3.62E-15 |
| F25B4.8 |  | down |  |  | 1.6 | 1.63E-10 |
| T24B8.3 |  | down |  |  | 1.6 | 1.26E-08 |
| F37B4.7 |  | down |  |  | 1.5 | 2.40E-14 |
| W07A12.8 |  | down | 1.6 | 1.18E-12 | 1.5 | 2.94E-13 |
| C17C3.12 | *acdh-2* | down |  |  | 1.5 | 7.55E-13 |
| Y11D7A.11 | *col-120* | down |  |  | 1.5 | 1.10E-10 |
| F28A12.4 |  | down |  |  | 1.5 | 2.88E-15 |
| T10H9.5 | *pmp-5* | down |  |  | 1.5 | 6.86E-14 |
| T15B7.1 |  | down |  |  | 1.5 | 1.15E-11 |
| F18E2.1 |  | down |  |  | 1.5 | 1.42E-16 |
| C48B4.1 |  | down |  |  | 1.5 | 1.48E-12 |
| C55B7.4 | *acdh-1* | down |  |  | 1.5 | 1.23E-13 |
| C55B7.4 | *acdh-1* | down |  |  | 1.5 | 5.55E-13 |
| F56A4.3 |  | down |  |  | 1.5 | 9.14E-12 |
| D1025.2 |  | down |  |  | 1.5 | 2.55E-14 |
| F08A8.3 |  | down |  |  | 1.5 | 5.28E-10 |
| ZK1058.6 | *nit-1* | down |  |  | 1.5 | 1.55E-08 |
| F58E6.4 |  | down |  |  | 1.5 | 2.10E-08 |
| T22B7.7 |  | down |  |  | 1.5 | 4.31E-08 |
| Y66D12A.13 |  | down |  |  | 1.5 | 2.06E-13 |
| C23H5.8 |  | down |  |  | 1.5 | 9.17E-10 |
| C25G4.6 |  | down |  |  | 1.5 | 3.39E-14 |
| T23F11.2 |  | down |  |  | 1.5 | 3.78E-12 |
| T04A8.5 |  | down |  |  | 1.5 | 3.70E-13 |
| C02B10.6 |  | down |  |  | 1.5 | 8.13E-12 |
