# Supplementary material for: Toxicogenomic analysis of Caenorhabditis elegans reveals novel genes and pathways involved in the resistance to cadmium toxicity
Source: Genome Biol. 2007 Jun 25;8(6):R122. doi: 10.1186/gb-2007-8-6-r122 (PMC2394766; doi:10.1186/gb-2007-8-6-r122)
Supplement: Additional data file 3 — Significantly enriched biological processes following 4 h and 24 h cadmium exposures and genes in the pathway that are cadmium-responsive. [file gb-2007-8-6-r122-S3.doc]

| **Enriched GO Category** | **Up Regulated** | | **Down Regulated** | | **Up Regulated** | | **Down Regulated** | |
| --- | --- | --- | --- | --- | --- | --- | --- | --- |
|  | **P values** | | | | **Gene Names** | | | |
|  | **4 h** | **24 h** | **4 h** | **24 h** | **4 h** | **24 h** | **4 h** | **24 h** |
| physiological process | 0.1006 | 0.0176 | 0.5819 | 0.043 | T08G5.10, ZC513.8, R05D8.9, T10B9.3, T01C3.4, T21D9.1, F41B5.3, ZK643.8, K04A8.5, T10B9.10, ZK742.3, T10B9.1, T10B9.2, F44C8.1, F46B3.17, AC3.7, C45H4.17, D2023.7, Y39B6A.24, C47A10.1, AC3.8, F41B5.2, B0213.15, F49H6.5, R04D3.1, M88.1, F49E12.10, F14F7.2, T18D3.3 | M88.1, W09G10.1, F46G10.6, T10B9.3, F36D3.9, F49H6.5, F43C11.8, ZK643.8, C47A10.1, F19C7.2, F10D2.11, F44C8.1, T10B9.1, ZC443.6, F14F7.2, F41B5.2, C06E4.3, T21E8.3, F46B6.8, F46B3.17, C08E3.7, Y39B6A.24, AC3.8, T10H4.12, C15C8.3, K08E7.9, F23B2.10, T10B9.10, ZK1251.2, K04A8.5, F41B5.7, F42A9.4, T21D9.1, F59D6.3, T08B1.3, Y51A2B.1, F27D9.2, D2023.7, R07B7.13, T01C3.4, B0213.15, F19C7.4, AC3.7, F28F8.2, PDB1.1, ZK455.4, R04D3.1, T10B9.2, T08G5.10, R05D8.9, T18D3.3, F42A9.5, F02D10.1, ZK742.3, ZC513.8, E03G2.2, F41B5.3 | ZK816.5 | ZK816.5, Y11D7A.11, F08A8.2, F28A12.4, F37B4.7, F58B3.3, ZK1058.6, D1025.2, F58B3.1, F58B3.2, R11G11.14, Y39G10AR.6, F49E12.9, F08A8.3, Y66D12A.13, C02B10.6, F52B11.4, R05F9.12, C48B4.1, Y4C6B.6, F17E9.11, T04A8.5, K09F5.2 |
| localization | 0 | 0.0002 | 1 | 0.0629 | C47A10.1, F41B5.2, ZC513.8, T10B9.3, B0213.15, T21D9.1, F41B5.3, T10B9.10, ZK742.3, T10B9.1, T10B9.2, F44C8.1, F46B3.17, R04D3.1, C45H4.17, F14F7.2, D2023.7, T18D3.3 | K08E7.9, W09G10.1, T10B9.10, T10B9.3, F41B5.7, F42A9.4, T21D9.1, C47A10.1, F27D9.2, F44C8.1, D2023.7, T10B9.1, B0213.15, F14F7.2, F41B5.2, T21E8.3, PDB1.1, T10B9.2, R04D3.1, T18D3.3, F46B3.17, F42A9.5, F02D10.1, ZK742.3, ZC513.8, E03G2.2, F41B5.3 |  | Y11D7A.11, F08A8.3, Y66D12A.13, F52B11.4, F08A8.2, F58B3.3, F37B4.7, C48B4.1, K09F5.2 |
| establishment of localization | 0 | 0.0002 | 1 | 0.1334 | C47A10.1, F41B5.2, ZC513.8, T10B9.3, B0213.15, T21D9.1, F41B5.3, T10B9.10, ZK742.3, T10B9.1, T10B9.2, F44C8.1, R04D3.1, F46B3.17, F14F7.2, C45H4.17, D2023.7, T18D3.3 | K08E7.9, W09G10.1, T10B9.10, T10B9.3, F41B5.7, F42A9.4, T21D9.1, C47A10.1, F27D9.2, F44C8.1, D2023.7, T10B9.1, B0213.15, F14F7.2, F41B5.2, T21E8.3, PDB1.1, R04D3.1, T10B9.2, T18D3.3, F46B3.17, F42A9.5, F02D10.1, ZK742.3, ZC513.8, F41B5.3, E03G2.2 |  | Y11D7A.11, F08A8.3, Y66D12A.13, F52B11.4, ,F08A8.2, F37B4.7, C48B4.1, K09F5.2 |
| transport | 0 | 0.0001 | 1 | 0.1161 | C47A10.1, F41B5.2, ZC513.8, T10B9.3, B0213.15, T21D9.1, F41B5.3, T10B9.10, ZK742.3, T10B9.1, T10B9.2, F44C8.1, F46B3.17, R04D3.1, C45H4.17, F14F7.2, D2023.7, T18D3.3 | K08E7.9, W09G10.1, T10B9.10, T10B9.3, F41B5.7, F42A9.4, C47A10.1, T21D9.1, F27D9.2, F44C8.1, D2023.7, T10B9.1, B0213.15, F14F7.2, F41B5.2, T21E8.3, PDB1.1, T10B9.2, R04D3.1, T18D3.3, F46B3.17, F42A9.5, F02D10.1, ZK742.3, ZC513.8, E03G2.2, F41B5.3 |  | Y11D7A.11, F08A8.3, Y66D12A.13, F52B11.4, F08A8.2, F37B4.7, C48B4.1, K09F5.2 |
| anion transport | 0.0062 | 0.0086 | 1 | 0.1448 | ZC513.8, F46B3.17, T21D9.1, D2023.7 | D2023.7, F46B3.17, W09G10.1, F02D10.1, T21D9.1, ZC513.8 |  | F52B11.4, Y11D7A.11 |
| inorganic anion transport | 0.0053 | 0.0074 | 1 | 0.1377 | ZC513.8, F46B3.17, T21D9.1, D2023.7 | D2023.7, F46B3.17, W09G10.1, F02D10.1, T21D9.1, ZC513.8 |  | F52B11.4, Y11D7A.11 |
| phosphate transport | 0.0035 | 0.0057 | 1 | 0.1261 | ZC513.8, F46B3.17, T21D9.1, D2023.7 | D2023.7, F46B3.17, W09G10.1, F02D10.1, T21D9.1, ZC513.8 |  | F52B11.4, Y11D7A.11 |
| metabolism | 0.0058 | 0.0051 | 0.3907 | 0.0342 | T08G5.10, R05D8.9, T10B9.3, T01C3.4, F41B5.3, ZK643.8, T10B9.10, K04A8.5, ZK742.3, T10B9.2, T10B9.1, F44C8.1, AC3.7, C45H4.17, D2023.7, Y39B6A.24, C47A10.1, AC3.8, F41B5.2, B0213.15, F49H6.5, R04D3.1, M88.1, F14F7.2, F49E12.10 | M88.1, F46G10.6, T10B9.3, F36D3.9, F49H6.5, F43C11.8, ZK643.8, C47A10.1, F19C7.2, F10D2.11, F44C8.1, T10B9.1, ZC443.6, F14F7.2, F41B5.2, C06E4.3, F46B6.8, C08E3.7, Y39B6A.24, AC3.8, T10H4.12, C15C8.3, F23B2.10, T10B9.10, K04A8.5, F41B5.7, F42A9.4, F59D6.3, T08B1.3, Y51A2B.1, D2023.7, R07B7.13, T01C3.4, B0213.15, F19C7.4, F28F8.2, AC3.7, ZK455.4, T10B9.2, R04D3.1, T08G5.10, R05D8.9, F42A9.5, ZK742.3, F41B5.3 | ZK816.5 | ZK816.5, F08A8.2, F28A12.4, F58B3.3, ZK1058.6, D1025.2, F58B3.1, F58B3.2, R11G11.14, Y39G10AR.6, F49E12.9, F08A8.3, C02B10.6, R05F9.12, C48B4.1, Y4C6B.6, F17E9.11, T04A8.5 |
| generation of precursor metabolites and energy | 0 | 0 | 1 | 0.1512 | T10B9.10, ZK742.3, F41B5.2, T10B9.3, T10B9.2, T10B9.1, F44C8.1, B0213.15, R04D3.1, F14F7.2, C45H4.17, F41B5.3 | F44C8.1, T10B9.1, B0213.15, F14F7.2, F41B5.2, T10B9.10, R04D3.1, T10B9.2, T10B9.3, F41B5.7, F42A9.4, F42A9.5, ZK742.3, F41B5.3 |  | C48B4.1, F08A8.3, F08A8.2 |
| electron transport | 0 | 0 | 1 | 0.0859 | T10B9.10, ZK742.3, F41B5.2, T10B9.3, T10B9.1, T10B9.2, F44C8.1, R04D3.1, B0213.15, F41B5.3, C45H4.17, F14F7.2 | F44C8.1, T10B9.1, B0213.15, F14F7.2, F41B5.2, T10B9.10, R04D3.1, T10B9.2, T10B9.3, F41B5.7, F42A9.5, F42A9.4, ZK742.3, F41B5.3 |  | C48B4.1, F08A8.3, F08A8.2 |
| proteolysis | 0.4902 | 0.0275 | 1 | 0.7531 | C47A10.1, Y39B6A.24 | T10H4.12, C15C8.3, F36D3.9, Y39B6A.24, C47A10.1, F59D6.3, F19C7.4, F19C7.2 |  | F28A12.4 |
| lipid metabolism | 0.0478 | 0.0202 | 1 | 0.0003 | K04A8.5, T08G5.10, T01C3.4 | F46B6.8, T08G5.10, K04A8.5, T01C3.4, ZK455.4 |  | F08A8.2, C48B4.1, Y4C6B.6, R11G11.14, F08A8.3 |
| cellular lipid metabolism | 1 | 0.4891 | 1 | 0.0001 |  | ZK455.4 |  | F08A8.2, C48B4.1, Y4C6B.6, F08A8.3 |
| catabolism | 0.2096 | 0.2479 | 1 | 0.0004 | T08G5.10, T01C3.4 | T08G5.10, T01C3.4, ZK455.4 |  | F58B3.3, F17E9.11, D1025.2, F58B3.1, F58B3.2 |
| cellular catabolism | 1 | 0.7476 | 1 | 0.0001 |  | ZK455.4 |  | F58B3.3, F17E9.11, D1025.2, F58B3.1, F58B3.2 |
| cellular carbohydrate catabolism | 1 | 1 | 1 | 0.0001 |  |  |  | F58B3.3, F17E9.11, F58B3.1, F58B3.2 |
| carbohydrate catabolism | 1 | 1 | 1 | 0.0001 |  |  |  | F58B3.3, F17E9.11, F58B3.1, F58B3.2 |
| carbohydrate metabolism | 1 | 1 | 1 | 0.0005 |  |  |  | R05F9.12, F58B3.3, F17E9.11, F58B3.1, F58B3.2 |
| cellular carbohydrate metabolism | 1 | 1 | 1 | 0.0011 |  |  |  | F58B3.3, F17E9.11, F58B3.1, F58B3.2 |
| carboxylic acid metabolism | 1 | 1 | 1 | 0.0014 |  |  |  | F08A8.2, D1025.2, C48B4.1, F08A8.3 |
| organic acid metabolism | 1 | 1 | 1 | 0.0014 |  |  |  | F08A8.2, D1025.2, C48B4.1, F08A8.3 |
| fatty acid metabolism | 1 | 1 | 1 | 0.0002 |  |  |  | F08A8.2, C48B4.1, F08A8.3 |
| fatty acid beta-oxidation | 1 | 1 | 1 | 0 |  |  |  | F08A8.2, C48B4.1, F08A8.3 |
| macromolecule catabolism | 1 | 1 | 1 | 0.0007 |  |  |  | F58B3.3, F17E9.11, F58B3.1, F58B3.2 |
| cellular macromolecule catabolism | 1 | 1 | 1 | 0.0005 |  |  |  | F58B3.3, F17E9.11, F58B3.1, F58B3.2 |
| cell wall catabolism | 1 | 1 | 1 | 0 |  |  |  | F58B3.3, F17E9.11, F58B3.1, F58B3.2 |
| peptidoglycan metabolism | 1 | 1 | 1 | 0 |  |  |  | F58B3.3, F17E9.11, F58B3.1, F58B3.2 |
| peptidoglycan catabolism | 1 | 1 | 1 | 0 |  |  |  | F58B3.3, F17E9.11, F58B3.1, F58B3.2 |

,
