# Supplementary material for: Toxicogenomic analysis of Caenorhabditis elegans reveals novel genes and pathways involved in the resistance to cadmium toxicity
Source: Genome Biol. 2007 Jun 25;8(6):R122. doi: 10.1186/gb-2007-8-6-r122 (PMC2394766; doi:10.1186/gb-2007-8-6-r122)
Supplement: Additional data file 4 — Significantly enriched molecular functions following 4 h and 24 h cadmium exposures and genes in the pathway that are cadmium-responsive. [file gb-2007-8-6-r122-S4.doc]

| **Enriched GO Category** | **Up Regulated** | | **Down Regulated** | | **Up Regulated** | | **Down Regulated** | |
| --- | --- | --- | --- | --- | --- | --- | --- | --- |
|  | **P values** | | | | **Gene Names** | | | |
|  | **4 h** | **24h** | **4 h** | **24 h** | **4 h** | **24 h** | **4 h** | **24 h** |
| Molecular function | 0.0014 | 0.0016 | 0.8071 | 0.0514 | R05D8.9, T18D3.3, R04D3.1, F42G8.8, T10B9.3, C45H4.17, F41B5.2, F49E12.10, T21D9.1, F13H6.3, T10B9.10, C12C8.1, D2023.7, F44C8.1, Y39B6A.24, Y37A1B.5, F14F7.2, T10B9.1, AC3.7, ZK643.8, T01C3.4, Y40B10A.7, T08G5.10, Y73C8C.2, F44E5.4, C47A10.1, K11G9.6, K04A8.5, Y40B10A.6, W01A11.1, ZC513.8, T10B9.2, M88.1, B0213.15, T26H2.5, F41B5.3, F49H6.5, ZK742.3, AC3.8, C31A11.5, F09B9.1 | F59D6.3, F14F7.2, F23B2.10, F28F8.2, Y39B6A.24, T10B9.10, F13H6.3, T10B9.3, Y73C8C.2, F41B5.7, F10D2.11, B0213.15, M88.1, K08E7.9, K04A8.5, K12D9.1, T01C3.4, AC3.8, R04D3.1, Y51A2B.1, T26H2.5, Y43F8A.3, F41B5.2, R12A1.4, AC3.7, F46G10.6, C17H1.9, F27D9.2, C01B10.10, C31A11.5, C08E3.7, F44C8.1, F08H9.9, T08G5.10, T21E8.3, F44E5.4, F46B6.8, F49H6.5, F19C7.2, T10H4.12, W01A11.1, T10B9.1, C47A10.1, F57B9.3, Y40B10A.7, M05D6.7, R07B7.13, C15C8.3, F02D10.1, ZC443.6, ZC513.8, C06E4.3, F42A9.5, PDB1.1, ZK643.8, K742.3, F43C11.8, T21D9.1, F36D3.9, T18D3.3, Y40B10A.6, T07C12.9, T08B1.3, T27F6.2, T10B9.2, T07H3.2, C12C8.1, E03G2.2, F09B9.1, K11G9.6, F42A9.4, R05D8.9, ZK455.4, ZK1251.2, D2023.7, F19C7.4, ZC204.12, F41B5.3, ZK666.7 | ZK816.5 | F17E9.11, F08A8.2, Y46C8AL.3, Y39G10AR.6, R05F9.12, T22B7.7, F18E2.1, F49E12.9, F28A12.4, F52B11.4, F37B4.7, T04A8.5, C48B4.1, F58B3.3, F58B3.2, C02B10.6, R11G11.14, F44G3.2, F08A8.3, ZK816.5, ZK1058.6, K09F5.2, C25G4.6, T09F5.9, Y66D12A.13, T15B7.1, B0218.6, Y11D7A.11, Y4C6B.6, F58B3.1 |
| Ion binding | 0 | 0.0001 | 1 | 1 | R04D3.1, T10B9.3, C45H4.17, T08G5.10, F41B5.2, C47A10.1, K11G9.6, T10B9.2, B0213.15, T10B9.10, T26H2.5, F41B5.3, F44C8.1, F49H6.5, F14F7.2, T10B9.1 | F23B2.10, F14F7.2, F42A9.4, T10B9.1, C47A10.1, T10B9.10, F42A9.5, F44C8.1, T08G5.10, T10B9.3, T10B9.2, R04D3.1, F41B5.7, F41B5.3, T26H2.5, F49H6.5, B0213.15, F43C11.8, K11G9.6, F41B5.2 |  |  |
| Metal ion binding | 0 | 0.0001 | 1 | 1 | R04D3.1, T10B9.3, C45H4.17, T08G5.10, F41B5.2, C47A10.1, K11G9.6, T10B9.2, B0213.15, T10B9.10, T26H2.5, F41B5.3, F44C8.1, F49H6.5, F14F7.2, T10B9.1 | F23B2.10, F14F7.2, F42A9.4, T10B9.1, C47A10.1, T10B9.10, F42A9.5, F44C8.1, T08G5.10, T10B9.3, T10B9.2, R04D3.1, F41B5.7, F41B5.3, T26H2.5, F49H6.5, B0213.15, F43C11.8, K11G9.6, F41B5.2 |  |  |
| Cation binding | 0 | 0 | 1 | 1 | R04D3.1, T10B9.3, C45H4.17, T08G5.10, F41B5.2, C47A10.1, K11G9.6, T10B9.2, B0213.15, T10B9.10, T26H2.5, F41B5.3, F44C8.1, F49H6.5, F14F7.2, T10B9.1 | F23B2.10, F14F7.2, F42A9.4, T10B9.1, C47A10.1, T10B9.10, F42A9.5, F44C8.1, T08G5.10, T10B9.3, T10B9.2, R04D3.1, F41B5.7, F41B5.3, T26H2.5, F49H6.5, B0213.15, F43C11.8, K11G9.6, F41B5.2 |  |  |
| Transition metal ion binding | 0 | 0 | 1 | 1 | R04D3.1, T10B9.3, C45H4.17, F41B5.2, C47A10.1, T10B9.2, B0213.15, T10B9.10, T26H2.5, F41B5.3, F44C8.1, F49H6.5, F14F7.2, T10B9.1 | F23B2.10, F14F7.2, F42A9.4, T10B9.1, C47A10.1, T10B9.10, F42A9.5, F44C8.1, T10B9.3, T10B9.2, R04D3.1, F41B5.7, F41B5.3, T26H2.5, F49H6.5, B0213.15, F43C11.8, F41B5.2 |  |  |
| Iron ion binding | 0 | 0 | 1 | 1 | R04D3.1, T10B9.3, C45H4.17, F41B5.2, T10B9.2, B0213.15, T10B9.10, F41B5.3, F44C8.1, F49H6.5, F14F7.2, T10B9.1 | F14F7.2, F42A9.4, T10B9.1, T10B9.10, F42A9.5, F44C8.1, T10B9.3, T10B9.2, R04D3.1, F41B5.7, F41B5.3, F49H6.5, B0213.15, F41B5.2 |  |  |
| Tetrapyrrole binding | 0 | 0 | 1 | 1 | T10B9.2, R04D3.1, B0213.15, T10B9.3, T10B9.10, C45H4.17, F41B5.3, F44C8.1, F41B5.2, F14F7.2, T10B9.1 | F14F7.2, F42A9.4, T10B9.1, T10B9.10, F42A9.5, F44C8.1, T10B9.3, T10B9.2, R04D3.1, F41B5.7, F41B5.3, B0213.15, F41B5.2 |  |  |
| Heme binding | 0 | 0 | 1 | 1 | T10B9.2, R04D3.1, B0213.15, T10B9.3, T10B9.10, C45H4.17, F41B5.3, F44C8.1, F41B5.2, F14F7.2, T10B9.1 | F14F7.2, F42A9.4, T10B9.1, T10B9.10, F42A9.5, F44C8.1, T10B9.3, T10B9.2, R04D3.1, F41B5.7, F41B5.3, B0213.15, F41B5.2 |  |  |
| Catalytic activity | 0 | 0 | 0.3354 | 0.0009 | R05D8.9, R04D3.1, F42G8.8, T10B9.3, C45H4.17, F41B5.2, F49E12.10, F13H6.3, T10B9.10, D2023.7, F44C8.1, Y39B6A.24, F14F7.2, T10B9.1, AC3.7, T01C3.4, Y40B10A.7, T08G5.10, C47A10.1, K04A8.5, Y40B10A.6, W01A11.1, T10B9.2, M88.1, B0213.15, F41B5.3, F49H6.5, ZK742.3, AC3.8, C31A11.5, F09B9.1 | F59D6.3, F14F7.2, F23B2.10, F28F8.2, Y39B6A.24, T10B9.10, F13H6.3, T10B9.3, F41B5.7, F10D2.11, B0213.15, M88.1, K08E7.9, K04A8.5, K12D9.1, T01C3.4, AC3.8, R04D3.1, Y51A2B.1, Y43F8A.3, F41B5.2, R12A1.4, AC3.7, C01B10.10, C31A11.5, F44C8.1, T08G5.10, T21E8.3, F46B6.8, F49H6.5, F19C7.2, T10H4.12, W01A11, T10B9.1, C47A10.1, F57B9.3, Y40B10A.7, M05D6.7, C15C8.3, ZC443.6, C06E4.3, F42A9.5, K742.3, F43C11.8, F36D3.9, Y40B10A.6, T07C12.9, T08B1.3, T10B9.2, C12C8.1, E03G2.2, F09B9.1, K11G9.6, F42A9.4, R05D8.9, ZK455.4, ZK1251.2, D2023.7, F19C7.4, ZC204.12, F41B5.3, ZK666.7 | ZK816.5 | F17E9.11, C02B10.6, R11G11.14, F44G3.2, F08A8.2, ZK816.5, F08A8.3, ZK1058.6, Y39G10AR.6, R05F9.12, T22B7.7, F18E2.1, F49E12.9, F28A12.4, T04A8.5, C48B4.1, Y4C6B.6, F58B3.1, F58B3.3, F58B3.2 |
| Oxidoreductase activity | 0 | 0 | 0.0447 | 0.0883 | R05D8.9, R04D3.1, ZK816.5, T10B9.3, C45H4.17, F41B5.2, T10B9.2, B0213.15, T10B9.10, F41B5.3, F44C8.1, ZK742.3, F14F7.2, T10B9.1 | F14F7.2, F42A9.4, R05D8.9, C06E4.3, T10B9.1, F42A9.5, T10B9.10, T08B1.3, F44C8.1, T10B9.3, T10B9.2, M05D6.7, R04D3.1, Y51A2B.1, F41B5.7, F41B5.3, ZK742.3, B0213.15, F41B5.2 | ZK816.5 | ZK816.5, F08A8.3, F08A8.2, C48B4.1 |
| Monooxygenase activity | 0 | 0 | 1 | 1 | T10B9.2, R04D3.1, B0213.15, T10B9.10, T10B9.3, F41B5.3, 5H4.17 | F14F7.2, F42A9.4, T10B9.1, F42A9.5, T10B9.10, F44C8.1, T10B9.3, T10B9.2, R04D3.1, F41B5.7, F41B5.3 |  |  |
|  |  |  |  |  | F44C8.1, F41B5.2, F14F7.2, T10B9.1 | B0213.15, F41B5.2 |  |  |
| Transferase activity, transferring one-carbon groups | 0.0496 | 0.0084 | 1 | 1 | Y40B10A.7, Y40B10A.6 | Y40B10A.7, K12D9.1, Y40B10A.6, T07C12.9 |  |  |
| Methyltransferase activity | 0.0488 | 0.0081 | 1 | 1 | Y40B10A.7, Y40B10A.6 | Y40B10A.7, K12D9.1, Y40B10A.6, T07C12.9 |  |  |
| Transferase activity, transferring hexosyl groups | 0.022 | 0.0172 | 1 | 0.4374 | M88.1, AC3.8, AC3.7 | ZC443.6, F10D2.11, AC3.8, AC3.7, M88.1 |  | Y39G10AR.6 |
| Transferase activity, transferring glycosyl groups | 0.0287 | 0.0246 | 1 | 0.1284 | M88.1, AC3.8, AC3.7 | ZC443.6, F10D2.11, AC3.8, AC3.7, M88.1 |  |  |
| Structural constituent of cuticle | 0.014 | 0.045 | 1 | 0.0908 | ZC513.8, D2023.7, T21D9.1 | ZC513.8, F02D10.1, D2023.7, T21D9.1 |  | F52B11.4, Y11D7A.11 |
| Carboxylic ester hydrolase activity | 0.0047 | 0.0055 | 1 | 0.2371 | T01C3.4, T08G5.10, K04A8.5 | K04A8.5, F46B6.8, T01C3.4, T08G5.10 |  | R11G11.14 |
| Hydrolase activity, acting on acid anhydrides | 0.7217 | 0.0918 | 1 | 1 | C47A10.1 | K08E7.9, C47A10.1, E03G2.2, F57B9.3, T21E8.3 |  |  |
| Peptidase activity | 0.5032 | 0.0286 | 1 | 0.7558 | Y39B6A.24, C47A10.1 | F59D6.3, F36D3.9, C15C8.3, Y39B6A.24, C47A10.1, F19C7.2, T10H4.12, F19C7.4 |  | F28A12.4 |
| ATPase activity | 0.5959 | 0.0301 | 1 | 1 | C47A10.1 | K08E7.9, C47A10.1, E03G2.2, F57B9.3, T21E8.3 |  |  |
| ATPase activity, coupled to movement of substances | 0.3813 | 0.0117 | 1 | 1 | C47A10.1 | K08E7.9, C47A10.1, E03G2.2, T21E8.3 |  |  |
| Carbohydrate binding | 0.6449 | 0.24 | 1 | 0.0139 | Y73C8C.2 | Y73C8C.2, ZK666.7, T27F6.2, F08H9.9 |  | Y46C8AL.3, B0218.6, T09F5.9, T15B7.1 |
| Sugar binding | 0.6181 | 0.2089 | 1 | 0.0113 | Y73C8C.2 | Y73C8C.2, ZK666.7, T27F6.2, F08H9.9 |  | Y46C8AL.3, B0218.6, T09F5.9, T15B7.1 |
| Hydrolase activity | 0.2755 | 0.0215 | 1 | 0.0015 | T01C3.4, F42G8.8, T08G5.10, Y39B6A.24, C47A10.1, K04A8.5, W01A11.1, | W01A11.1, F36D3.9, F59D6.3, Y39B6A.24, C47A10.1, F57B9.3, C15C8.3, E03G2.2, K08E7.9, K04A8.5, T01C3.4, ZK455.4, T21E8.3, T08G5.10, F19C7.4, F46B6.8, F19C7.2, T10H4.12 |  | F18E2.1, F17E9.11, C02B10.6, R11G11.14, F28A12.4, F58B3.1, Y4C6B.6, ZK1058.6, F58B3.3, R05F9.12, F58B3.2 |
| Hydrolase activity, acting on glycosyl bonds | 1 | 1 | 1 | 0 |  |  |  | F58B3.1, Y4C6B.6, F17E9.11, F58B3.3, R05F9.12, F58B3.2 |
| Hydrolase activity, hydrolyzing O-glycosyl compounds | 1 | 1 | 1 | 0 |  |  |  | F58B3.1, Y4C6B.6, F17E9.11, F58B3.3, R05F9.12, F58B3.2 |
| Lysozyme activity | 1 | 1 | 1 | 0 |  |  |  | F58B3.1, F17E9.11, F58B3.3, F58B3.2 |

,
