# Supplementary material for: Toxicogenomic analysis of Caenorhabditis elegans reveals novel genes and pathways involved in the resistance to cadmium toxicity
Source: Genome Biol. 2007 Jun 25;8(6):R122. doi: 10.1186/gb-2007-8-6-r122 (PMC2394766; doi:10.1186/gb-2007-8-6-r122)
Supplement: Additional data file 5 — Cadmium-responsive genes tested in the first round RNAi screen. [file gb-2007-8-6-r122-S5.doc]

| Target Gene Name | CGC Gene Name | Fold Induction (24 h) | Second Round (yes/no) |
| --- | --- | --- | --- |
| F35E8.11 | *cdr-1* | 111.4 | Y |
| R04D3.1 | *cyp-14A4* | 32.4 | N |
| T08G5.10 | *mtl-2* | 31.7 | Y |
| Y46G5A.24 |  | 18.3 | Y |
| T26H2.5 |  | 15.2 | Y |
| K11G9.6 | *mtl-1* | 15.0 | Y |
| F28D1.4 | *thn-3* | 14.3 | Y |
| Y39B6A.24 |  | 11.4 | N |
| F28D1.3 | *thn-1* | 9.9 | N |
| AC3.7 | *ugt-1* | 8.2 | Y |
| C02A12.1 | *gst-33* | 7.8 | N |
| T10B9.1 | *cyp-13A4* | 7.1 | Y |
| F08F8.5 |  | 7.1 | N |
| F41B5.2 | *cyp-33C7* | 6.9 | Y |
| T08G5.1 |  | 6.8 | N |
| T16G1.6 |  | 6.1 | Y |
| F53C3.12 |  | 5.7 | Y |
| C08E3.6 |  | 5.2 | Y |
| F35E8.8 | *gst-38* | 5.0 | Y |
| T10B9.2 | *cyp-13A5* | 4.4 | Y |
| T18D3.3 |  | 4.3 | N |
| C27H5.4 |  | 4.1 | Y |
| Y40B10A.6 |  | 3.8 | N |
| F28D1.5 | *thn-2* | 3.7 | N |
| T01C3.4 |  | 3.7 | N |
| Y40B10A.7 |  | 3.5 | Y |
| F49F1.6 |  | 3.4 | Y |
| F15E11.12 |  | 3.4 | Y |
| W08A12.4 |  | 3.3 | Y |
| R05D8.9 |  | 3.2 | N |
| K04A8.5 |  | 3.0 | Y |
| ZC196.6 |  | 3.0 | Y |
| C08E3.10 |  | 3.0 | Y |
| Y73C8C.2 |  | 3.0 | Y |
| T08E11.1 |  | 2.9 | Y |
| C54D10.8 |  | 2.9 | N |
| C17H1.4 |  | 2.9 | Y |
| Y39G8B.7 |  | 2.8 | N |
| C45G7.3 |  | 2.8 | N |
| F26F2.3 |  | 2.7 | N |
| F37B1.8 | *gst-19* | 2.7 | Y |
| ZK742.3 |  | 2.7 | N |
| T07D10.4 | *clec-15* | 2.6 | Y |
| C08E3.1 |  | 2.4 | N |
| F37B1.1 | *gst-24* | 2.4 | N |
| T10B9.3 | *cyp-13A6* | 2.4 | Y |
| W01A11.1 |  | 2.3 | Y |
| C47A10.1 | *pgp-9* | 2.3 | N |
| F42C5.3 |  | 2.3 | Y |
| B0024.4 |  | 2.3 | Y |
| Y105C5A.12 |  | 2.3 | N |
| T27E4.2 | *hsp-16.11* | 2.3 | Y |
| F15E6.8 |  | 2.2 | N |
| F13H6.3 |  | 2.2 | N |
| K02E2.7 |  | 2.2 | N |
| F57B9.3 |  | 2.2 | Y |
| T16G1.5 |  | 2.2 | N |
| F49H6.5 |  | 2.2 | Y |
| M88.1 | *ugt-62* | 2.2 | Y |
| F53H2.1 |  | 2.2 | Y |
| B0284.2 |  | 2.1 | N |
| C54D10.7 |  | 2.1 | N |
| F56C3.9 |  | 2.1 | N |
| T27F6.2 | *clec-12* | 2.1 | Y |
| D2023.7 | *col-158* | 2.1 | N |
| C29F7.2 |  | 2.1 | Y |
| T12D8.5 |  | 2.1 | Y |
| F41B5.3 | *cyp-33C5* | 2.1 | Y |
| F15A4.8 |  | 2.0 | Y |
| T28D9.3 |  | 2.0 | Y |
| F09B9.1 |  | 2.0 | Y |
| Y75B8A.28 |  | 2.0 | N |
| F15E11.1 |  | 2.0 | N |
| B0284.4 |  | 2.0 | N |
| F47H4.10 | *skr-5* | 2.0 | N |
| K09D9.1 |  | 2.0 | Y |
| C29F3.7 |  | 1.9 | N |
| F59B1.8 |  | 1.9 | Y |
| T21E8.3 | *pgp-8* | 1.8 | N |
| Y46H3A.3 | *hsp-16.2* | 1.7 | Y |
| R05F9.5 | *gst-9* | 1.7 | Y |
| C12C8.1 | *hsp-70* | 1.6 | N |
| Y46H3A.2 | *hsp-16.41* | 1.6 | Y |
| F44E5.4 |  | 1.6 | Y |
| R07B7.13 | *nhr-206* | 1.6 | N |
| Y39E4A.2 | *ttm-1* | 1.6 | Y |
| PDB1.1 |  | 1.5 | N |
| K01D12.11 | *cdr-4* | 1.5 | Y |
| K08E7.9 | *pgp-1* | 1.5 | N |
| F37B1.4 | *gst-15* | 1.5 | N |
| ZK1251.2 | *ins-7* | 1.5 | N |
| E03G2.2 | *mrp-3* | 1.5 | N |
| F46G10.6 | *mxl-3* | 1.5 | N |
