# Supplementary material for: Toxicogenomic analysis of Caenorhabditis elegans reveals novel genes and pathways involved in the resistance to cadmium toxicity
Source: Genome Biol. 2007 Jun 25;8(6):R122. doi: 10.1186/gb-2007-8-6-r122 (PMC2394766; doi:10.1186/gb-2007-8-6-r122)
Supplement: Additional data file 6 — Effect of RNAi and cadmium on C. elegans body size in the second round RNAi screen using COPAS BioSort. [file gb-2007-8-6-r122-S6.doc]

| **Category** | **Target Gene** | **CGC**  **Gene Name** | **24h Fold**  **Induction** | **Mean Length ^a^** | | **Diff. Means** | **p-value ^b^** |
| --- | --- | --- | --- | --- | --- | --- | --- |
|  |  |  |  | **Cd-** | **Cd+** |  |  |
| Strong | F09B9.1 |  | 2 | 0.97 | 0.45 | 0.52 | p<1.0E-16 |
|  | M88.1 | *ugt-62* | 2.2 | 1.01 | 0.54 | 0.47 | p<1.0E-16 |
|  | C17H1.4 |  | 2.9 | 1.01 | 0.62 | 0.39 | p<1.0E-16 |
|  | T27F6.2 | *clec-12* | 2.1 | 1.02 | 0.65 | 0.37 | p<1.0E-16 |
|  | F42C5.3 |  | 2.3 | 0.97 | 0.60 | 0.37 | p<1.0E-16 |
|  | F53H2.1 |  | 2.2 | 1.00 | 0.63 | 0.37 | p<1.0E-16 |
|  | F35E8.11 | *cdr-1* | 111.4 | 1.01 | 0.65 | 0.35 | p<1.0E-16 |
|  | F49H6.5 |  | 2.2 | 0.99 | 0.65 | 0.34 | p<1.0E-16 |
|  | Y46G5A.24 |  | 18.3 | 1.06 | 0.73 | 0.33 | p<1.0E-16 |
|  | F44E5.4 |  | 1.6 | 0.99 | 0.66 | 0.33 | p<1.0E-16 |
|  | F28D1.4 | *thn-3* | 14.3 | 1.05 | 0.72 | 0.33 | p<1.0E-16 |
| Medium | W01A11.1 |  | 2.3 | 0.98 | 0.67 | 0.31 | p<1.0E-16 |
|  | T10B9.3 | *cyp-13A6* | 2.4 | 1.02 | 0.71 | 0.31 | p<1.0E-16 |
|  | C08E3.10 |  | 3 | 1.00 | 0.69 | 0.31 | p<1.0E-16 |
|  | Y73C8C.2 |  | 3 | 1.00 | 0.70 | 0.30 | p<1.0E-16 |
|  | F15E11.12 |  | 3.4 | 0.99 | 0.69 | 0.30 | p<1.0E-16 |
|  | F15A4.8 |  | 2 | 1.01 | 0.71 | 0.30 | p<1.0E-16 |
|  | F41B5.3 | *cyp-33C5* | 2.1 | 1.02 | 0.73 | 0.29 | p<1.0E-16 |
|  | Y39E4A.2 | *ttm-1* | 1.6 | 0.89 | 0.60 | 0.29 | p<1.0E-16 |
|  | Y46H3A.3 | *hsp-16.2* | 1.7 | 1.02 | 0.74 | 0.28 | p<1.0E-16 |
|  | T12D8.5 |  | 2.1 | 0.97 | 0.69 | 0.28 | p<1.0E-16 |
|  | Y40B10A.7 |  | 3.5 | 0.99 | 0.72 | 0.27 | p<1.0E-16 |
|  | Y46H3A.2 | *hsp-16.41* | 1.6 | 1.02 | 0.75 | 0.27 | p<1.0E-16 |
|  | T26H2.5 |  | 15.2 | 1.01 | 0.75 | 0.26 | p<1.0E-16 |
|  | K01D12.11 | *cdr-4* | 1.5 | 1.01 | 0.76 | 0.25 | p<1.0E-16 |
|  | T10B9.1 | *cyp-13A4* | 7.1 | 0.88 | 0.63 | 0.25 | p<1.0E-16 |
|  | F59B1.8 |  | 1.9 | 1.03 | 0.78 | 0.25 | p<1.0E-16 |
|  | T07D10.4 | *clec-15* | 2.6 | 0.99 | 0.75 | 0.24 | p<1.0E-16 |
|  | K09D9.1 |  | 2 | 0.95 | 0.71 | 0.24 | p<1.0E-16 |
|  | ZC196.6 |  | 3 | 0.98 | 0.74 | 0.24 | p<1.0E-16 |
|  | T28D9.3 |  | 2 | 1.00 | 0.76 | 0.24 | p<1.0E-16 |
|  | F49F1.6 |  | 3.4 | 0.99 | 0.76 | 0.23 | p<1.0E-16 |
|  | F41B5.2 | *cyp-33C7* | 6.9 | 0.97 | 0.74 | 0.23 | p<1.0E-16 |
|  | T08G5.10 | *mtl-2* |  | 1.01^c^ | 0.79^c^ | 0.22 | p<1.0E-16 |
|  | C08E3.6 |  | 5.2 | 1.01 | 0.79 | 0.22 | p<1.0E-16 |
|  | K04A8.5 |  | 3 | 0.99 | 0.78 | 0.21 | p<1.0E-16 |
|  | W08A12.4 |  | 3.3 | 0.95 | 0.74 | 0.21 | p<1.0E-16 |
|  | C29F7.2 |  | 2.1 | 0.97 | 0.76 | 0.21 | p<1.0E-16 |
|  | T27E4.2 | *hsp-16.11* | 2.3 | 1.01 | 0.81 | 0.20 | p<1.0E-16 |
| Weak | K11G9.6 | *mtl-1* | 15 | 0.92 | 0.74 | 0.18 | p<1.0E-16 |
|  | R05F9.5 | *gst-9* | 1.7 | 0.93 | 0.76 | 0.17 | p<1.0E-16 |
|  | F37B1.8 | *gst-19* | 2.7 | 0.98 | 0.81 | 0.17 | p<1.0E-16 |
|  | B0024.4 |  | 2.3 | 0.98 | 0.82 | 0.16 | p<1.0E-16 |
|  | T10B9.2 | *cyp-13A5* | 4.4 | 0.98 | 0.82 | 0.16 | 1.11E-16 |
|  | AC3.7 | *ugt-1* | 8.2 | 1.02 | 0.86 | 0.16 | 5.40E-12 |
|  | T16G1.6 |  | 6.1 | 1.01 | 0.86 | 0.15 | 5.60E-13 |
|  | F35E8.8 | *gst-38* | 5 | 1.01 | 0.89 | 0.12 | 2.22E-12 |
|  | F53C3.12 |  | 5.7 | 1.00 | 0.89 | 0.11 | 1.22E-08 |
|  | T08E11.1 |  | 2.9 | 0.99 | 0.91 | 0.08 | 3.63E-04 |
|  | C27H5.4 |  | 4.1 | 0.97 | 0.89 | 0.08 | 2.92E-07 |

^a^ The mean body length was normalized by dividing the mean body length of nematodes exposed to the control RNAi to those with the target gene knocked-down (n = 200-500).

^b^ Welch two-sample t-tests were applied for significance tests.

^c^ RNAi of *mtl-2* was conducted in an *mtl-1* null mutant, *mtl-1* (tm1770).
