# Supplementary material for: Toxicogenomic analysis of Caenorhabditis elegans reveals novel genes and pathways involved in the resistance to cadmium toxicity
Source: Genome Biol. 2007 Jun 25;8(6):R122. doi: 10.1186/gb-2007-8-6-r122 (PMC2394766; doi:10.1186/gb-2007-8-6-r122)
Supplement: Additional data file 7 — Primer sequences used in qRT-PCR. [file gb-2007-8-6-r122-S7.doc]

| **Gene** | **Forward** | **Reverse** |
| --- | --- | --- |
| *mlc-2* | TTGACAGGAACTGACCCAGAGG | ATAGCCTTGACCTCATCCTCG |
| *cdr-1* | TCTTCTCTCAATTGGCAACTG | TTTGGGTAAACTTCATGACGA |
| *mtl-1* | TGGATGTAAGGGAGACTGCAA | CATTTTAATGAGCCGCAGCA |
| *mtl-2* | AAGTGTGCCAACTGCGAATGT | GCTTTCAAGAAAAAACCTCGA |
| *hsp-70* | TGAAATTGAAGCAAAGGACAA | TGTGGATAATTGCTGGAATGG |
| *gst-38* | TGATTTGCTGGGACGTGAAA | CTGGACGAGTCTCAATCCATT |
| T24H10.7 | TTTCGTTTCGTCAACAACCG | AATCCACCGTCTGAATCGTCA |
